# Accelerometer-Measured Physical Activity, Fitness and Indicators of Cardiometabolic Risk among Rural Adolescents: A Cross-Sectional Study at 15-Year Follow-up of the MINIMat Cohort

**DOI:** 10.1007/s44197-024-00245-1

**Published:** 2024-05-21

**Authors:** Mohammad Redwanul Islam, Christine Delisle Nyström, Maria Kippler, Eero Kajantie, Marie Löf, Syed Moshfiqur Rahman, Eva-Charlotte Ekström

**Affiliations:** 1 Department of Women’s and Children’s Health, Uppsala University, Uppsala, Sweden; 2 Department of Biosciences and Nutrition, Karolinska Institutet, Huddinge, Sweden; 3 Institute of Environmental Medicine, Unit of Metals and Health, Karolinska Institutet, Stockholm, Sweden; 4 Department of Public Health and Welfare, Finnish Institute for Health and Welfare, Helsinki, Finland; 5 PEDEGO Research Unit, MRC Oulu, Oulu University Hospital & University of Oulu, Oulu, Finland; 6 Department of Health, Medicine and Caring Sciences, Linköping University, Linköping, Sweden; 7 Maternal and Child Health Division, International Centre for Diarrhoeal Disease Research, Bangladesh (icddr,b), Dhaka, Bangladesh

**Keywords:** Physical activity, Adolescents, Bangladesh, Blood pressure, Waist circumference, Triglyceride, Insulin resistance

## Abstract

**Background:**

Little is known about the relationship of physical activity (PA) and fitness with cardiometabolic risk among rural adolescents in low- and middle-income countries. Thus, we examined the associations of PA and fitness with selected cardiometabolic indicators along with potential gender-based differences in a birth cohort of rural adolescents from southeast Bangladesh.

**Methods:**

We utilized data from the 15-year follow-up of Maternal and Infant Nutrition Interventions in Matlab (MINIMat) cohort (*n* = 2253). Wrist-worn ActiGraph wGT3x-BT accelerometers were used to estimate sedentary time (ST) and PA. Fitness was assessed using: handgrip strength, standing long jump, and Chester Step Test. Anthropometric parameters, systolic blood pressure (SBP), and fasting lipid, insulin and glucose levels were measured. We calculated insulin resistance using the Homeostasis Model Assessment equation (HOMA-IR). Linear regression and isotemporal substitution models were fitted.

**Results:**

The adolescents spent 64 min/day (inter-quartile range: 50–81) in moderate-to-vigorous physical activity (MVPA). A 10-minute-per-day higher vigorous PA (VPA) was associated with: 4.9% (95% confidence interval (CI): 2.9–6.8%) lower waist circumference (WC), 3.2 mmHg (95% CI: 1.5–4.8) lower SBP, 10.4% (95% CI: 2.9–17.3%) lower TG, and 24.4% (95% CI: 11.3–34.9%) lower HOMA-IR. MVPA showed similar associations of notably smaller magnitude. Except for WC, the associations were more pronounced among the boys. Substituting ST with VPA of equal duration was associated with lower WC, SBP, triglyceride and HOMA-IR. Grip strength was favorably associated with all indicators, displaying considerably large effect sizes.

**Conclusion:**

Our findings indicated beneficial roles of PA– particularly VPA– and muscular fitness in shaping cardiometabolic profile in mid-adolescence. VPA and grip strength may represent potential targets for preventive strategies tailored to adolescents in resource-limited settings.

**Supplementary Information:**

The online version contains supplementary material available at 10.1007/s44197-024-00245-1.

## Background

While the trend of increasing body mass index (BMI) among adolescents has plateaued at a high level in many high-income countries, it continues to escalate in low- and middle-income countries (LMICs) in south and southeast Asia [1]. This increase in BMI coincides with a rapid nutrition transition in LMICs that involves marked declines in habitual physical activity (PA) and increasing sedentary behavior at population level [2]. A recent analysis of self-reported data from 1.6 million adolescents across 146 countries [3] demonstrates the global prevalence of insufficient PA (i.e., less than 60 min of moderate-to-vigorous PA daily) to be 81%. When disaggregated by country income, the prevalences of insufficient PA among adolescents in low- and lower-middle-income countries appear to be 84.9% and 79.3%, respectively. This study also indicates an entrenched gender differential globally, with insufficient PA being more prevalent among girls than boys [3].

Sufficient PA during adolescence promotes healthy weight status, improved muscular and cardiorespiratory fitness, and better cardiometabolic health [4]. Adverse cardiometabolic risk profile emerging in adolescence predicts hypertension, type 2 diabetes, dyslipidemias and cardiovascular events in the adulthood [5, 6]. In addition, PA behaviors established during adolescence tend to track well into adulthood [7]. Therefore, exploring the relationship of PA with indicators of cardiometabolic risk during adolescence is of public health interest. However, our understanding of this relationship remains limited for several reasons. Empirical evidence on the association of PA with cardiometabolic risk markers has been derived mostly from studies among adults [8]. Unlike adulthood, adolescence is a transitional phase characterized by dynamic growth and maturation [9] as well as socio-behavioral transitions that influence the adoption of PA behaviors [10]. Consequently, the findings from studies among adults are not readily applicable to adolescents. Moreover, the vast majority of the studies on PA and cardiometabolic risk are from high-income countries [8], even though LMICs in South Asia host about 350 million adolescents– the highest of any global region [11]. A significant proportion of these adolescents has been exposed to early-life undernutrition [12] potentially increasing their susceptibility to cardiometabolic disorders in ensuing adulthood [13]. Accelerometer-measured PA data from adolescents in low-income settings are extremely sparse [14]. Self-reports of PA provide limited validity for estimating total PA and are prone to under-estimation of sedentary time (ST) owing to recall and social desirability biases [15]. Additionally, moderate-to-vigorous physical activity (MVPA) and ST display stronger associations with such indicators as systolic blood pressure (SBP), insulin resistance (IR) and fasting lipids, when measured objectively compared to self-reports [16, 17]. Thus, there remains a need of objective assessment of PA in free-living conditions in resource-limited LMIC settings.

Health benefits of physical fitness– a phenotype related to PA– are widely recognized. Among adults, each five-kilogram decrease in handgrip strength increases the hazard for all-cause and cardiovascular mortality by 16–17% [18]. Longitudinal studies have linked adolescents’ fitness level to reduced all-cause premature mortality [19] and improved cardiometabolic profile in adulthood [20]. Nevertheless, when it comes to fitness and conventional cardiometabolic risk markers, studies have generated divergent findings: from no association [21, 22] to unanticipated positive associations of muscular fitness with composite risk score and SBP [22, 23]. Whether this resulted from methodological heterogeneity, inadequate control of confounding or context-specific variations remains elusive. Moreover, few studies utilized a combination of reliable fitness tests in large population-based sample of adolescents from resource-limited settings [20, 24]. Hence, in a birth cohort of rural adolescents from Bangladesh, we sought to examine the associations of (i) accelerometer-measured ST and time spent in intensity-specific PA, and (ii) measures of fitness (handgrip strength, standing long jump and aerobic capacity) with: waist circumference (WC), SBP, plasma triglyceride (TG), total cholesterol (TC), low- and high-density lipoproteins (LDL and HDL), and IR along with potential difference in associations by gender.

## Methods

### Study Design, Participants and Setting

**Fig. 1:**
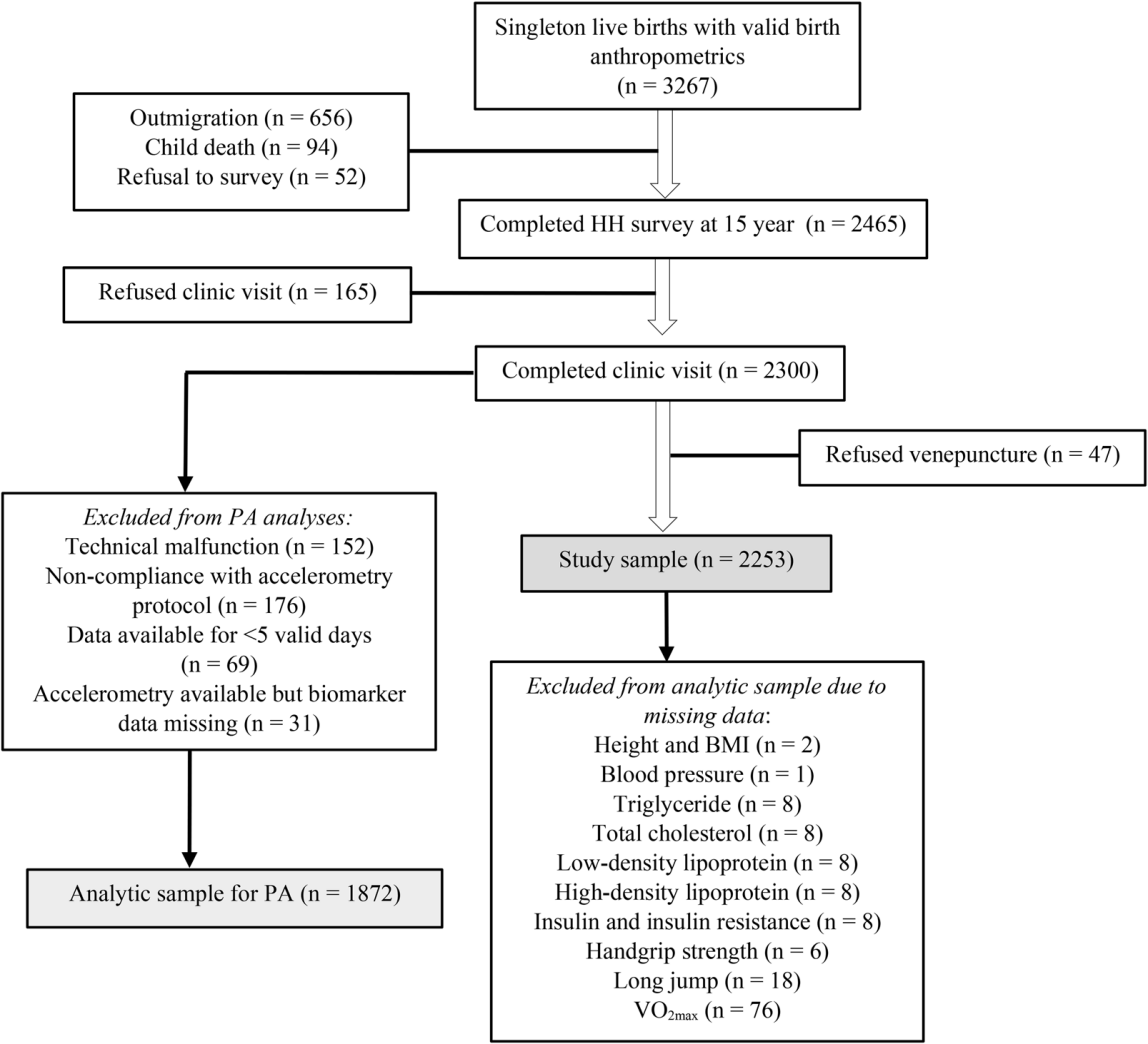
Flowchart for inclusion of MINIMat adolescents into the present study. *Abbreviations* HH, household; PA, physical activity; VO_2max_, maximal oxygen consumption

This cross-sectional study utilized data collected during the 15-year follow-up of the MINIMat (Maternal and Infant Nutrition Interventions in Matlab) trial from September 2017 to June 2019. MINIMat (reg#ISRCTN16581394) was a community-based, randomized trial that tested the effects of prenatal food and micronutrient supplementation on maternal and birth outcomes [25]. Between 2001 and 2003, a total of 4436 pregnant women from Matlab were randomized. This resulted in 3267 singleton live births with valid birth anthropometrics, forming the MINIMat cohort that has been intensively followed up [26]. The latest, 15-year follow-up comprised three parts: formative phase, household survey and clinic visit. The eligibility criteria for the follow-up were: (i) being born as singletons to the mothers randomized in the trial, and (ii) having valid birth anthropometrics available. Trained interviewers with at least 12 years of formal education interviewed the adolescent-mother/guardian dyads at their houses using a pre-tested, structured questionnaire. The clinic visit involved anthropometric and physical fitness assessments as well as collection of fasting blood samples. These were carried out by trained nurses, following a standardized protocol [27–29]. Out of the 3267 eligible adolescents, 2465 (75.5%) completed the household survey and 2300 (70.4%) completed the clinic visit. The participant flow into the present study and reasons for loss to follow-up are shown in Fig. 1 (Results section ).

Matlab is a rural sub-district, located about 55 km to the southeast of the capital city of Dhaka. The community is agrarian and rice farming is the main occupation in Matlab, but a few villages rely on fishing as the means of income [30].

### Assessment of Physical Activity through Accelerometry

Wrist-mounted, triaxial ActiGraph wGT3X-BT accelerometers (ActiGraph Corp, USA) with a dynamic range of ± 8 G were used to assess PA in free-living conditions. After the clinic visit, the participants were fitted with pre-tested accelerometers on the non-dominant wrist, and instructed to keep wearing the devices for at least five consecutive 24-hour periods (including night-time), except during water-based activities (e.g., swimming and bathing). The devices were initialized to start collecting data from 12:00pm on the day they were fitted. The sampling frequency was 90 Hz. Data were processed and analyzed in ActiLife software (version 6.13.4) in five-second epochs with the default ‘Normal filter’ [31] option. The five-second epoch suits the duration of PA bouts commonly observed among adolescents [32], and provides sufficient resolution to avoid under-estimation of ST [33] and VPA [31]. Non-wear time (defined as ≥ 60 min of consecutive zero counts allowing non-zero interruptions of up to 2 min) and sleep time were deducted using algorithms validated among adolescents [34, 35]. A valid day entailed an awake wear time of ≥ 600 min, and participants accumulating at least five such valid days– including one weekend day– were retained in the analysis. For wrist-worn, tri-axial accelerometers; vector magnitude (VM, the square root of the sum of squared activity counts from the three axes) is more appropriate than the vertical axis counts [36]. We employed cut-points based on VM counts per epoch, validated by Chandler et al., to determine sedentary time (≤ 305) and time engaged in intensity-specific PA: light-intensity (LIPA: 306–817), moderate (MPA: 818–1968), vigorous (VPA: ≥1969), and moderate-to-vigorous (MVPA: ≥818) [36]. Additionally, total PA was assessed in terms of daily aggregate of VM (i.e., VM counts per minute divided by the number of valid days).

### Assessment of Physical Fitness

We assessed the muscular and cardiorespiratory components of fitness through: (i) handgrip strength, an indicator of upper body isometric strength; (ii) distance obtained from standing long jump, a proxy for lower body explosive strength; and (iii) aerobic capacity or maximal oxygen consumption (VO_2max_) from a submaximal, multistage test– Chester Step Test (CST) [28, 29, 37].

A digital, handgrip dynamometer (TKK 5401 Grip-D, Takei Scientific Instruments Company, Japan; range: 5–100 kg; accuracy: ±2 kg) was used for measuring handgrip strength. The dynamometer’s grip-span was adjusted to the hand size of the participant. The adolescents stood with their arms completely extended and squeezed the dynamometer gradually to the maximum of their strength for at least 2 s [38]. The test was performed twice on each hand, alternating between the right and left side. The dynamometer was reset to zero after each attempt. The handgrip strength (kg) was derived as the mean of the highest readings from the right and left hands. To adjust for differences in body mass, weight-normalized grip strength was calculated by dividing handgrip strength with body weight (kg) [39, 40].

The standing long jump test [28] was conducted indoors on a non-slip, hard surface. The adolescents were instructed to stand behind a 50-cm long take-off line with their feet shoulder width apart, push off vigorously and jump as far forward as possible to land on both feet staying upright. The distance (cm) between the line and the heel mark of the foot closest to the line was measured. The maximum distance obtained from two attempts was used in the analysis.

The CST involved stepping on to and off a step, 30-cm high, at a rate set by a metronome– commencing with 15 steps/minute and increasing every two minutes by five steps/minute– with a heart rate (HR) monitor strapped to chest [29]. The test continued until the adolescents reached 80% of the predicted maximum HR (i.e., 164/minute), reported perceived exertion of 15 (“Hard”) on the Borg scale [41], or completed all the five stages of the test. VO_2max_ (mL/kg/minute) was predicted using the CST software that incorporates a statistical line of best fit across the incremental HR recordings.

### Assessment of Cardiometabolic Risk Indicators

Body weight was recorded with a digital scale (Tanita BC-418 Body Composition Analyzer, 0.2 kg) and height with a stadiometer (Seca 214, 0.1 cm) while adolescents wore standard light clothes provided by the project and were barefoot. The weight of the clothes (200 g) was deducted from the measured weight. Body mass index (BMI) was calculated by dividing body weight with height squared (kg/m^2^). BMI-for-age z-score (BAZ) was calculated using the World Health Organization reference [42]. For descriptive purpose, we categorized the adolescents as thin (BAZ < − 2), normal-weight (− 2 ≤ BAZ ≥ + 1), and overweight/obese (BAZ > + 1). WC was measured midway between the lower margin of the least palpable rib and iliac crest with a non-elastic tape (TALC) to the nearest 0.1 cm. Sitting BP was measured in triplicates, at two-minute intervals, using an Omron M10 device after a 10-minute seated rest. The arithmetic mean of the second and third readings [43] was used for analysis. Venous blood samples (6 mL) after an overnight fast were collected in Lithium-heparin tubes (Sarstedt). The samples were centrifuged; plasma was separated, aliquoted and stored at − 70 °C. The frozen blood samples were transported from Bangladesh to Sweden using an insulated packaging system where dry ice (solid carbon dioxide) served as the coolant to maintain the temperature. Plasma TC, TG, LDL and HDL levels were measured in a Cobas Analyzer (Roche) through enzymatic colorimetric assay at the laboratory of the Department of Clinical Chemistry, Skåne University Hospital, Sweden. Plasma insulin level was measured at the International Centre for Diarrhoeal Disease Research, Bangladesh (icddr, b) laboratory. Fasting glucose was measured using the Contour TS Blood Glucose Monitoring System (Bayer) during the clinic visit. We calculated IR using the Homeostasis Model Assessment (HOMA) equation: HOMA-IR = (fasting insulin mU/L × fasting glucose in mmol/L) ÷ 22.5 [44].

### Socio-Demographic Variables

Gender was a dichotomous variable (girl/boy). An asset score was calculated for each household from principal component analysis [45] of the data on ownership of a set of durables (e.g., mobile phone, television, refrigerator, etcetera), access to electricity and sanitary latrine, and nature of fuel used. We constructed a categorical variable (household wealth) by converting asset scores into tertiles: the lowest, intermediate and highest tertiles representing the poorest, middle-status and richest households, respectively. Educational status was categorized according to completed mw of formal education: none, primary (1–5 years), and secondary (6–12 years) for adolescents or secondary and above (≥ 6 years) for mothers.

### Statistical Analysis

Categorical variables are described with frequency and percentage, and continuous variables with mean and standard deviation (SD) or median and interquartile range. We checked the distributions of the continuous variables using histograms and quantile-quantile plots. Three right-skewed variables were natural log (Ln) transformed: WC, TG and HOMA-IR. The PA variables used in analyses of associations were: ST and time engaged in intensity-specific PA (all in minutes/day), and total PA. The total PA variable was converted into tertiles. Those with handgrip strength and VO_2max_ greater than five SD of corresponding mean (*n* = 2 for handgrip, *n* = 6 for VO_2max_) were flagged implausible and excluded from the analyses. For analyses of associations, the weight-normalized grip strength was used. The CST software excluded adolescents without adequate HR data points (at least two, *n* = 70) from the prediction of VO_2max_. At bivariate level, gender differences were evaluated with Chi-squared test, independent samples t-test or Wilcoxon rank-sum test. Linear regression models– for all participants and by gender– were fitted, and regression coefficients with 95% confidence intervals (CI) are reported. We examined quantile-quantile plots of the residuals and residuals versus fitted plots to rule out violation of assumptions. The adjusted models accounted for gender (except when gender-stratified), household wealth and maternal education (directed acyclic graph presented in Supplementary Fig. 1). Models with ST and intensity-specific and total PA as exposures were also adjusted for awake wear time. We did not adjust for BMI, considering it a mediator in the relationship of PA and fitness with cardiometabolic indicators [46, 47]. Multiplicative interaction terms incorporating gender were tested and the following were found statistically significant: MPA × gender (*P* < 0.001) and MVPA × gender (*P* < 0.001) for HDL; weight-normalized grip strength × gender for WC (*P* < 0.001) and SBP (*P* = 0.002); standing long jump × gender for TG (*P* = 0.004). Further, we fitted isotemporal substitution models as described by Mekary et al. [48, 49] to examine the effect of substituting ST with intensity-specific PA (for instance, with LIPA or MPA or VPA) for the same amount of time. Given the concern related to multi-collinearity in isotemporal substitution model [50], we examined variance inflation factor (VIF); and no values exceeded 2.6 (Supplementary Table 2). All tests were two-tailed and P-values < 0.05 were considered statistically significant. The analyses were performed in R, version 4.2.2 [51].

### Ethics Approval

The 15-year follow-up has been approved by the Ethical Review Committee at icddr, b in Dhaka, Bangladesh (PR–17029; date 2017/05/23). An additional approval has been obtained from the Ethics Review Authority (*Etikprövningsmyndigheten*) in Sweden (2021–02796; date 2021/11/15). We obtained written informed consent from the mothers and assent from the adolescents. The study was carried out in accordance with the Declaration of Helsinki.

## Results

Of the 3267 eligible adolescents, 2465 completed the household survey. The clinic visit was refused by 165 adolescents and another 47 refused venepuncture. Thus, the study sample comprised 2253 adolescents. Based on availability of valid accelerometry data and corresponding data on cardiometabolic indicators, 1872 adolescents were retained in the analyses involving PA. Adolescents with missing data on height, and hence, BMI (*n* = 2); blood pressure (*n* = 1); TG (*n* = 8); TC (*n* = 8); LDL (*n* = 8); HDL (*n* = 8); insulin, and hence, HOMA-IR (*n* = 8); handgrip strength (*n* = 6); long jump (*n* = 18); and VO_2max_ (*n* = 76) were excluded from analyses involving the respective variables (Fig. 1).

### Characteristics of the Participants

The socio-demographic and anthropometric characteristics along with accelerometry, fitness and cardiometabolic profiles of the participants are shown in Table 1. The boys were taller and heavier than the girls; but the median BMI was higher among the girls. Overweight/obesity was more prevalent among the girls than the boys (7.9% versus 6.2%), but thinness was more common among the boys (28.2% versus 11.8%). Approximately 40% of the girls belonged to the poorest households, while 28.5% of the boys came from the poorest households.

The sample median for daily awake wear time exceeded 15 h (approximately 937 min/day). Valid accelerometry data were available for five (10.6%), six (77%), seven (10%), eight (2.2%), nine (0.1%), and 10 (0.1%) days. The total daily PA varied by gender: the majority of the girls (36.1%) belonged to the lowest tertile, whereas the majority of the boys (38.2%) belonged to the highest tertile. The medians of daily vector magnitude counts per minute (CPM) across these tertiles were: 256 (interquartile range: 231.8–275.8) for the lowest tertile, 320 (305.6–335.1) for the intermediate tertile, and 395 (371.2–437.6) for the highest tertile. The adolescents spent about 69% of the daily awake wear time sedentary and about 7% in MVPA. On average, the girls spent more time sedentary and in MPA per day, whereas the boys spent more time in LIPA and in VPA per day. The boys recorded higher levels of handgrip strength, standing long jump distance and VO_2max_ than the girls. The girls presented higher levels of fasting plasma lipids (except HDL) and insulin resistance, while the boys had higher SBP (Table 1).

**Table 1:** Descriptive characteristics of the study participants

Characteristics	All (*n* = 2253)		Boys (*n* = 1079)		Girls (*n* = 1174)		*P* ^1^
	*n*	Value	*n*	Value	*n*	Value	
Age (years)	2253	15.0 (0.1)	1079	15.0 (0.1)	1174	15.0 (0.1)	0.382
Height (cm)	2251	156.5 (7.6)	1078	160.5 (7.7)	1173	152.9 (5.4)	**< 0.001**
Weight (kg)	2253	43.9 (39.3–49.7)	1079	45.3 (39.3–51.0)	1174	43.1 (39.2–48.5)	**< 0.001**
BMI (kg/m^2^)	2251	17.8 (16.3–19.8)	1078	17.2 (15.9–18.9)	1173	18.4 (16.9–20.5)	**< 0.001**
BAZ categories^**2**^	2251						**< 0.001**
Thin	442	19.6	304	28.2	138	11.8	
Normal	1649	73.3	707	65.6	942	80.3	
Overweight/obese	160	7.1	67	6.2	93	7.9	
Household wealth	2253						**< 0.001**
Poorest	775	34.4	308	28.5	467	39.8	
Intermediate	723	32.1	397	36.8	326	27.8	
Richest	755	33.5	374	34.7	381	32.4	
Maternal education	2253						**0.027**
None	447	19.8	210	19.5	237	20.2	
Primary	800	35.5	357	33.1	443	37.7	
Secondary or above	1006	44.7	512	47.4	494	42.1	
Adolescent education	2253						**< 0.001**
None	355	15.8	214	19.8	141	12.0	
Primary	57	2.5	45	4.2	12	1.0	
Secondary	1841	81.7	820	76.0	1021	87.0	
*Accelerometry characteristics*							
Number of valid days	1872	6.0 (0.6)	887	6.0 (0.5)	985	6.0 (0.6)	0.254
Awake wear time (min/day)	1872	936.6 (874.6–991.7)	887	933.8 (871.1–991.0)	985	938.8 (877.7–992.0)	0.399
Total daily PA	1872						**< 0.001**
Tertile 1	624	33.3	269	30.3	355	36.1	
Tertile 2	625	33.4	279	31.5	346	35.1	
Tertile 3	623	33.3	339	38.2	284	28.8	
ST (min/day)	1872	641.2 (92.0)	887	635.3 (93.0)	985	646.5 (90.9)	**0.008**
ST percentage^**3**^	1872	69.1 (65.1–73.2)	887	68.6 (64.4–72.9)	985	69.4 (65.9–73.4)	**0.002**
LIPA (min/day)	1872	220.5 (42.4)	887	224.3 (45.1)	985	217.2 (39.4)	**< 0.001**
Moderate PA (min/day)	1872	64.6 (22.6)	887	62.9 (23.0)	985	66.2 (22.2)	**0.001**
Vigorous PA (min/day)	1872	1.0 (0.4–2.6)	887	2.4 (1.1–4.8)	985	0.5 (0.2–0.9)	**< 0.001**
Vigorous PA percentage	1872	0.1 (0.04–0.3)	887	0.3 (0.1–0.5)	985	0.05 (0.03–0.1)	**< 0.001**
MVPA (min/day)	1872	64.3 (49.9–80.9)	887	63.8 (48.1–81.5)	985	64.5 (51.2–80.4)	0.270
MVPA percentage^**3**^	1872	6.9 (5.4–8.7)	887	6.8 (5.3–8.7)	985	7.0 (5.6–8.6)	0.363
*Fitness characteristics*							
Handgrip strength (kg)	2247	24.4 (21.1–29.8)	1078	30.0 (25.3–33.7)	1169	21.7 (20.0–24.0)	**< 0.001**
Weight-normalized grip strength^**4**^	2247	0.58 (0.12)	1078	0.65 (0.10)	1169	0.51 (0.09)	**< 0.001**
Standing long jump distance (cm)	2235	141.0 (124.0–162.0)	1071	162.0 (148.0–175.0)	1164	125.8 (115.8–136.2)	**< 0.001**
VO_2max_ (mL/kg/min)	2177	34.5 (31.0–37.3)	1057	35.1 (31.8–38.2)	1120	33.8 (30.3–36.6)	**< 0.001**
*Cardiometabolic indicators*							
WC (cm)	2253	62.4 (58.9–66.8)	1079	62.4 (59.3–66.4)	1174	62.4 (58.8–67.2)	0.989
SBP (mm of Hg)	2252	108.8 (8.0)	1078	110.3 (8.2)	1174	107.4 (7.6)	**< 0.001**
TG (mmol/L)	2245	0.9 (0.7–1.2)	1077	0.9 (0.7–1.1)	1168	1.0 (0.8–1.3)	**< 0.001**
TC (mmol/L)	2245	3.6 (0.7)	1077	3.4 (0.6)	1168	3.7 (0.7)	**< 0.001**
LDL (mmol/L)	2245	2.1 (0.6)	1077	2.0 (0.5)	1168	2.2 (0.6)	**< 0.001**
HDL (mmol/L)	2245	1.0 (0.2)	1077	1.0 (0.2)	1168	1.0 (0.2)	0.441
Insulin (µU/L)	2245	11.4 (7.8–18.3)	1078	9.6 (6.6–16.3)	1167	12.9 (9.3–20.2)	**< 0.001**
Glucose (mmol/L)	2253	5.2 (0.5)	1079	5.3 (0.5)	1174	5.1 (0.4)	**< 0.001**
HOMA-IR	2245	2.6 (1.7–4.2)	1078	2.2 (1.5–3.8)	1167	2.9 (2.1–4.7)	**< 0.001**

*Abbreviations* BMI, body mass index; BAZ, BMI-for-age z-score; PA, physical activity; CPM, counts per minute; ST, sedentary time; LIPA, light-intensity physical activity; MVPA, moderate-to-vigorous physical activity; VO_2max_, maximal oxygen consumption; WC, waist circumference; SBP, systolic blood pressure; TG, triglyceride; TC, total cholesterol; LDL, low-density lipoprotein; HDL, high-density lipoprotein; HOMA-IR, insulin resistance from Homeostasis Model Assessment. Values represent percentage for categorical variables, mean with standard deviation for continuous variables (approximately) normally distributed, or median with inter-quartile range for continuous variables that were skewed. Missing data: height and BMI (*n* = 2), handgrip strength and weight-normalized grip strength (*n* = 6), standing long jump (*n* = 18), VO_2max_ (*n* = 76), SBP (*n* = 1), TG (*n* = 8), TC (*n* = 8), LDL (*n* = 8), HDL (*n* = 8), and insulin and HOMA-IR (*n* = 8)

^**1**^P value for gender difference from Chi-squared test, independent samples t-test, or Wilcoxon rank-sum test

^**2**^BAZ below − 2 SD and above + 1 SD define thinness and overweight/obesity, respectively

^**3**^Percentage of daily awake wear time spent sedentary and in moderate-to-vigorous physical activity

^**4**^Derived by dividing participant’s handgrip strength (kg) by his/her body weight (kg)

### Association of Total PA with the Cardiometabolic Indicators

Small, statistically significant differences in predicted mean levels between the top and bottom tertiles were observed for WC and IR. Adolescents in the top tertile of total PA had approximately 1.1 cm lower WC than their peers in the bottom tertile (*P* = 0.004). On gender stratification, the difference in IR between the top and bottom tertiles lost statistical significance among the girls (*P* = 0.323), but remained significant among the boys (*P* = 0.004). Compared to boys in the bottom tertile, boys in the top tertile had about 1.5 mmHg lower SBP (*P* = 0.027) and 0.06 mmol/L lower plasma TG (*P* = 0.048). Total PA showed no association with TC, LDL and HDL (Table 2).

**Table 2:** Estimated levels of the selected cardiometabolic indicators by tertiles of total daily physical activity among the adolescents

Indicator	Tertile 1 of total daily PA (VMCPM < 289.9)	Tertile 2 of total daily PA (VMCPM 289.9–350.1)	Tertile 3 of total daily PA (VMCPM > 350.1)	*P*-value^2^	*P*-value^3^
	Predicted^1^ mean (95% CI)	Predicted^1^ mean (95% CI)	Predicted^1^ mean (95% CI)		
*Entire sample*					
Waist circumference^**4**^ cm	63.8 (63.3, 64.4)	63.8 (63.2, 64.3)	62.7 (62.2, 63.3)	0.833	**0.004**
Systolic blood pressure (mmHg)	108.9 (108.3, 109.6)	109.2 (108.6, 109.9)	108.3 (107.7, 108.9)	0.501	0.173
Triglyceride^**4**^ mmol/L	0.97 (0.94, 1.01)	0.95 (0.92, 0.98)	0.95 (0.92, 0.98)	0.293	0.273
Total cholesterol mmol/L	3.60 (3.55, 3.66)	3.57 (3.52, 3.62)	3.57 (3.52, 3.63)	0.319	0.395
Low-density lipoprotein mmol/L	2.16 (2.11, 2.21)	2.12 (2.08, 2.17)	2.14 (2.10, 2.19)	0.257	0.545
High-density lipoprotein mmol/L	1.02 (1.00, 1.03)	1.03 (1.01, 1.04)	1.02 (1.00, 1.04)	0.418	0.700
HOMA-IR^**4,5**^	3.02 (2.85, 3.21)	2.91 (2.74, 3.08)	2.70 (2.54, 2.87)	0.340	**0.008**
*Girls*					
Waist circumference^**4**^ cm	63.8 (63.0, 64.5)	63.8 (63.1, 64.6)	62.6 (61.8, 63.4)	0.921	**0.038**
Systolic blood pressure (mmHg)	106.9 (106.1, 107.8)	108.0 (107.1, 108.8)	107.2 (106.3, 108.1)	0.079	0.697
Triglyceride^**4**^ mmol/L	1.03 (0.99, 1.08)	1.03 (0.99, 1.07)	1.05 (1.00, 1.10)	0.902	0.639
Total cholesterol mmol/L	3.76 (3.68, 3.83)	3.72 (3.65, 3.80)	3.75 (3.66, 3.83)	0.490	0.862
Low-density lipoprotein mmol/L	2.29 (2.22, 2.35)	2.24 (2.17, 2.30)	2.30 (2.23, 2.37)	0.298	0.790
High-density lipoprotein mmol/L	1.02 (0.99, 1.04)	1.03 (1.00, 1.05)	0.99 (0.97, 1.02)	0.550	0.194
HOMA-IR^**4,5**^	3.29 (3.05, 3.55)	3.37 (3.13, 3.64)	3.11 (2.86, 3.38)	0.637	0.323
*Boys*					
Waist circumference^**4**^ cm	63.9 (63.1, 64.8)	63.6 (62.9, 64.4)	62.8 (62.1, 63.6)	0.591	**0.044**
Systolic blood pressure (mmHg)	111.0 (110.0, 112.1)	110.4 (109.4, 111.4)	109.5 (108.6, 110.4)	0.387	**0.027**
Triglyceride^**4**^ mmol/L	0.93 (0.88, 0.98)	0.88 (0.84, 0.92)	0.87 (0.83, 0.91)	0.126	**0.048**
Total cholesterol mmol/L	3.45 (3.38, 3.53)	3.41 (3.34, 3.48)	3.40 (3.33, 3.46)	0.398	0.266
Low-density lipoprotein mmol/L	2.04 (1.97, 2.10)	2.01 (1.95, 2.07)	1.98 (1.93, 2.04)	0.531	0.224
High-density lipoprotein mmol/L	1.01 (0.98, 1.04)	1.02 (1.00, 1.05)	1.04 (1.02, 1.07)	0.491	0.074
HOMA-IR^**4,5**^	2.84 (2.58, 3.13)	2.51 (2.29, 2.75)	2.36 (2.17, 2.57)	0.059	**0.004**

*Abbreviations* PA, physical activity; VMCPM, vector magnitude counts per minute; CI, confidence interval; HOMA-IR, Homeostasis Model Assessment-insulin resistance. Total daily physical activity was based on vector magnitude counts per minute accumulated per day

^**1**^Predicted from multivariable linear regression model adjusted for gender (when not stratified by gender), household wealth, maternal education, and awake wear time (min/day)

^**2**^For the difference between tertile 1 and tertile 2

^**3**^For the difference between tertile 1 and tertile 3

^**4**^For these three right-skewed variables, the predicted values represent geometric mean

^**5**^Additionally adjusted for sample storage time, that showed a negative correlation with fasting insulin level (Spearman’s ρ = − 0.2, *P* < 0.001)

*P*-values in bold indicate statistically significant differences in mean

### Associations of ST and Intensity-Specific PA with the Cardiometabolic Indicators

Table 3 presents the unstandardized regression coefficients with 95% CIs from adjusted linear models for each 10-minute change in ST and time spent in MPA, VPA and MVPA. Each 10-minute increase in ST per day was associated with 0.1% (95% CI: 0.04–0.2%; *P* = 0.005) higher WC and 0.9% (95% CI: 0.3–1.5%; *P* = 0.004) higher IR. When stratified by gender, the positive association of ST with WC remained statistically significant among the girls (β_adjusted_ for Ln WC: 0.001; 95% CI: 0.0002–0.003; *P* = 0.023); whereas that with IR slightly strengthened and remained statistically significant among the boys (β_adjusted_ for Ln HOMA-IR: 0.013; 95% CI: 0.004–0.022; *P* = 0.006). ST also displayed a small, positive association with SBP among the boys (β_adjusted_: 0.1; 95% CI: 0.05–0.25; *P* = 0.002).

Time spent in MVPA was negatively associated with WC and IR. A 10-minute per day higher MVPA was associated with: 0.4% (95% CI: 0.2–0.6%; *P* < 0.001) lower WC and 1.9% (95% CI: 0.4–3.3%; *P* = 0.012) lower IR. When stratified by gender, the negative association of MVPA with IR was not statistically significant among the girls (*P* = 0.307). Among the boys, 10-minutes per day higher MVPA resulted in: 0.3 mmHg (95% CI: 0.1–0.5; *P* = 0.006) lower SBP, 2.7% (95% CI: 0.5–4.7%; *P* = 0.015) lower IR, and 0.01 mmol/L (95% CI: 0.007–0.02; *P* < 0.001) higher HDL.

Apart from TC and LDL, time spent in VPA showed statistically significant associations with all other indicators. Each 10-minute-per-day increase in VPA was associated with: 4.9% (95% CI: 2.9–6.8%; *P* < 0.001) lower WC, 3.2 mmHg (95% CI: 1.5–4.8; *P* < 0.001) lower SBP, 10.4% (95% CI: 2.9–17.3%; *P* = 0.008) lower TG, 24.4% (95% CI: 11.3–34.9%; *P* < 0.001) lower IR, and 0.1 mmol/L (95% CI: 0.06–0.15; *P* < 0.001) higher HDL. While its negative association with WC strengthened, VPA showed no statistically significant association with any other indicator among the girls (Table 3).

**Table 3:** Associations of time spent sedentary and in moderate, vigorous and moderate-to-vigorous physical activity with the selected cardiometabolic indicators per 10-minute change

Indicator	Time spent sedentary (minutes/day)		Time spent in MPA (minutes/day)		Time spent in VPA (minutes/day)		Time spent in MVPA (minutes/day)	
	β (95% CI)	*P*-value	β (95% CI)	*P*-value	β (95% CI)	*P*-value	β (95% CI)	*P*-value
*Entire sample*								
Ln waist circumference^1,2^	**0.001 (0.0004, 0.002)**	**0.005**	**−0.003 (− 0.006, − 0.001)**	**0.001**	**−0.05 (− 0.07, − 0.03)**	**< 0.001**	**−0.004 (− 0.006, − 0.002)**	**< 0.001**
Systolic blood pressure^2^	0.06 (− 0.01, 0.12)	0.085	−0.16 (− 0.32, 0.01)	0.065	**−3.17 (− 4.82, − 1.52)**	**< 0.001**	**−0.17 (− 0.33, − 0.01)**	**0.034**
Ln triglyceride^1,2^	0.002 (− 0.001, 0.005)	0.193	−0.004 (− 0.012, 0.004)	0.334	**−0.11 (− 0.19, − 0.03)**	**0.008**	−0.005 (− 0.013, 0.003)	0.240
Total cholesterol^2^	0.001 (− 0.005, 0.006)	0.762	0.0003 (− 0.013, 0.014)	0.964	0.02 (− 0.12, 0.15)	0.815	0.0004 (− 0.012, 0.013)	0.948
Low-density lipoprotein^2^	0.001 (− 0.003, 0.006)	0.610	0.0001 (− 0.011, 0.012)	0.983	−0.02 (− 0.13, 0.10)	0.773	−0.00004 (− 0.011, 0.011)	0.994
High-density lipoprotein^2^	**−0.002 (− 0.004, − 0.0003)**	**0.024**	**0.006 (0.001, 0.01)**	**0.018**	**0.10 (0.06, 0.15)**	**< 0.001**	**0.006 (0.002, 0.01)**	**0.007**
Ln HOMA-IR^1,2,3^	**0.009 (0.003, 0.015)**	**0.004**	**−0.018 (− 0.033, − 0.002)**	**0.023**	**−0.28 (− 0.43, − 0.12)**	**< 0.001**	**−0.019 (− 0.033, − 0.004)**	**0.012**
*Girls*								
Ln waist circumference^1,2^	**0.001 (0.0002, 0.003)**	**0.023**	**−0.004** **(− 0.007, − 0.0005)**	**0.023**	**−0.09 (− 0.17, − 0.01)**	**0.035**	**−0.004** **(− 0.007, − 0.0005)**	**0.021**
Systolic blood pressure^2^	−0.05 (− 0.14, 0.04)	0.296	0.01 (− 0.21, 0.23)	0.917	−2.32 (− 8.21, 3.58)	0.440	0.01 (− 0.21, 0.23)	0.942
Ln triglyceride^1,2^	0.0004 (− 0.004, 0.005)	0.875	−0.00002 (− 0.011, 0.011)	0.997	0.10 (− 0.19, 0.40)	0.497	0.0001 (− 0.011, 0.011)	0.983
Total cholesterol^2^	0.002 (− 0.006, 0.010)	0.633	−0.004 (− 0.024, 0.017)	0.721	−0.09 (− 0.62, 0.45)	0.748	−0.004 (− 0.024, 0.016)	0.717
Low-density lipoprotein^2^	0.0001 (− 0.007, 0.007)	0.985	0.002 (− 0.016, 0.02)	0.820	−0.15 (− 0.61, 0.32)	0.531	0.002 (− 0.016, 0.019)	0.843
High-density lipoprotein^2^	0.0005 (− 0.002, 0.003)	0.691	−0.002 (− 0.009, 0.004)	0.532	0.03 (− 0.14, 0.20)	0.736	−0.002 (− 0.01, 0.004)	0.548
Ln HOMA-IR^1,2,3^	0.005 (− 0.003, 0.001)	0.215	−0.01 (− 0.03, 0.001)	0.310	−0.19 (− 0.73, 0.35)	0.492	−0.01 (− 0.03, 0.01)	0.307
*Boys*								
Ln waist circumference^1,2^	0.001 (− 0.0002, 0.002)	0.092	**−0.003** **(− 0.007, − 0.0005)**	**0.024**	**−0.05 (− 0.07, − 0.02)**	**< 0.001**	**−0.004 (− 0.007, − 0.001)**	**0.009**
Systolic blood pressure^2^	**0.15 (0.05, 0.25)**	**0.002**	**−0.32 (− 0.57, − 0.07)**	**0.012**	**−3.08 (− 4.87, − 1.29)**	**< 0.001**	**−0.32 (− 0.55, − 0.09)**	**0.006**
Ln triglyceride^1,2^	0.004 (− 0.001, 0.008)	0.121	−0.01 (− 0.02, 0.004)	0.184	**−0.12 (− 0.21, − 0.03)**	**0.007**	−0.01 (− 0.02, 0.002)	0.117
Total cholesterol^2^	−0.0003 (− 0.007, 0.007)	0.922	0.004 (− 0.014, 0.022)	0.631	0.04 (− 0.09, 0.17)	0.586	0.004 (− 0.012, 0.02)	0.608
Low-density lipoprotein^2^	0.002 (− 0.004, 0.008)	0.458	−0.002 (− 0.017, 0.013)	0.791	0.004 (− 0.10, 0.11)	0.945	−0.002 (− 0.016, 0.012)	0.814
High-density lipoprotein^2^	**−0.004 (− 0.007,−0.002)**	**< 0.001**	**0.014 (0.007, 0.02)**	**< 0.001**	**0.11 (0.06, 0.15)**	**< 0.001**	**0.013 (0.007, 0.02)**	**< 0.001**
Ln HOMA-IR^1,2,3^	**0.013 (0.004, 0.022)**	**0.006**	**−0.02 (− 0.05, − 0.002)**	**0.03**	**−0.30 (− 0.47, − 0.13)**	**< 0.001**	**−0.027 (− 0.048, − 0.005)**	**0.015**

*Abbreviations* MPA, moderate physical activity; VPA, vigorous physical activity; MVPA, moderate-to-vigorous physical activity; CI, confidence interval; HOMA-IR, insulin resistance from Homeostasis Model Assessment. β represents unstandardized regression coefficient. ^**1**^Ln represents natural-log transformed outcome variables where the base of the log was 2.71828. ^**2**^Adjusted for gender (when not stratified by gender), household wealth, maternal education and awake wear time (minutes/day). ^**3**^Additionally adjusted for sample storage time as it showed a negative correlation with fasting insulin level (Spearman’s ρ = − 0.2, *P* < 0.001). Statistically significant coefficients are presented in bold

The results from the isotemporal substitution model, presented in Supplementary Table 1, showed that substituting 10 min per day of ST with 10 min per day of VPA was associated with lower WC, SBP, TG and IR, and higher HDL. No statistically significant associations were observed for TC and LDL when similarly replacing ST with VPA. Substitution of ST with MPA was not associated with any indicator. Substitution of ST with LIPA was associated with lower IR. None of the VIF values for the isotemporal substitution models exceeded 2.6, ruling out substantial collinearity among the accelerometry variables (Supplementary Table 2).

### Association of Physical Fitness with the Cardiometabolic Indicators

The adjusted linear regression analyses for associations of weight-normalized grip strength, standing long jump and VO_2max_ with the cardiometabolic indicators are presented in Table 4. After adjusting for gender, household wealth and maternal education, a higher weight-normalized grip strength was associated with lower WC (β_adjusted_ for Ln WC: −0.589; 95% CI: −0.628, − 0.550), TG (β_adjusted_ for Ln TG: −0.590; 95% CI: −0.769, − 0.410), TC (β_adjusted_: −0.59; 95% CI: −0.89, − 0.30), LDL (β_adjusted_: −0.53; 95% CI: −0.78, − 0.28) and IR (β_adjusted_ for Ln HOMA-IR: −1.56; 95% CI: −1.89, − 1.23). A unitary increase in weight-normalized grip strength was associated with 0.3 mmol/L (95% CI: 0.17–0.37) higher HDL. These associations remained statistically significant after adjusting for ST and MVPA. There was no association between weight-normalized grip strength and SBP among the boys, whereas a unitary increase was associated with 16 mmHg (95% CI: 11.4–21.4) lower SBP. This gender-specific, negative association was further strengthened after adjusting for ST and MVPA.

Statistically significant, negative associations were observed between standing long jump and levels of TC (β_adjusted_: −0.03; 95% CI: −0.04, − 0.01) and LDL (β_adjusted_: −0.02; 95% CI: −0.03, − 0.009). These associations were independent of gender, household wealth and maternal education; and remained significant after additionally adjusting for ST and MVPA. Among the girls, a 10-cm higher long jump distance was associated with 0.7% (95% CI: 0.3–1.1%) lower WC. Among the boys, a 10-cm higher long jump distance was associated with 1.9% (95% CI: 0.6–3.0%) lower TG. These gender-specific associations also remained significant after additionally adjusting for ST and MVPA (Table 4).

VO_2max_ was negatively associated with WC after controlling for gender, household wealth, maternal education, ST and MVPA. When stratified by gender, the negative association remained statistically significant only among the boys. VO_2max_ was not associated with any other indicator (Table 4).

**Table 4:** Associations of weight-normalized handgrip strength, standing long jump (per 10-centimeter increase) and maximal oxygen consumption with the selected cardiometabolic indicators

Indicator	Weight-normalized grip strength^a^		Standing long jump (cm)		Maximal oxygen consumption (mL/kg/min)	
	β (95% CI)	*P*-value	β (95% CI)	*P*-value	β (95% CI)	*P*-value
*Entire sample*						
*Ln waist circumference* ^*b*^						
Adjusted model 1^**c**^	**−0.589 (− 0.628, − 0.550)**	**< 0.001**	**−0.003 (− 0.005, − 0.001)**	**0.009**	**−0.001 (− 0.002, − 0.0002)**	**0.010**
Adjusted model 2^**d**^	**−0.604 (− 0.647, − 0.560)**	**< 0.001**	**−0.003 (− 0.006, − 0.001)**	**0.011**	**−0.001 (− 0.002, − 0.0003)**	**0.010**
*Systolic blood pressure*						
Adjusted model 1^**c, e**^	**−9.5 (− 13.1, − 6.0)**	**< 0.001**	−0.1 (− 0.2, 0.1)	0.483	−0.04 (− 0.10, 0.02)	0.206
Adjusted model 2^**d, e**^	**−9.7 (− 13.6, − 5.8)**	**< 0.001**	−0.1 (− 0.3, 0.1)	0.462	−0.02 (− 0.09, 0.04)	0.508
*Ln triglyceride* ^*b*^						
Adjusted model 1^**c**^	**−0.590 (− 0.769, − 0.410)**	**< 0.001**	−0.008 (− 0.017, 0.001)	0.09	−0.001 (− 0.004, 0.002)	0.514
Adjusted Model 2^**d**^	**−0.599 (− 0.794, − 0.403)**	**< 0.001**	**−0.011 (− 0.021, − 0.001)**	**0.034**	−0.001 (− 0.004, 0.003)	0.693
*Total cholesterol*						
Adjusted model 1^**c**^	**−0.59 (− 0.89, − 0.30)**	**< 0.001**	**−0.03 (− 0.04, − 0.01)**	**< 0.001**	−0.001 (− 0.006, 0.004)	0.796
Adjusted model 2^**d**^	**−0.63 (− 0.95, − 0.31)**	**< 0.001**	**−0.03 (− 0.04, − 0.01)**	**0.001**	−0.002 (− 0.007, 0.004)	0.502
*Low-density lipoprotein*						
Adjusted model 1^**c**^	**−0.53 (− 0.78, − 0.28)**	**< 0.001**	**−0.02 (− 0.03, − 0.009)**	**0.001**	−0.001 (− 0.005, 0.003)	0.718
Adjusted model 2^**d**^	**−0.55 (− 0.83, − 0.28)**	**< 0.001**	**−0.02 (− 0.03, − 0.007)**	**0.003**	−0.002 (− 0.006, 0.003)	0.497
*High-density lipoprotein*						
Adjusted model 1^**c**^	**0.27 (0.17, 0.37)**	**< 0.001**	0.0003 (− 0.005, 0.005)	0.907	0.001 (− 0.0002, 0.003)	0.094
Adjusted model 2^**d**^	**0.26 (0.15, 0.37)**	**< 0.001**	0.002 (− 0.004, 0.008)	0.504	0.001 (− 0.0007, 0.003)	0.198
*Ln HOMA-IR* ^*b*^						
Adjusted model 1^**c, f**^	**−1.56 (− 1.89, − 1.23)**	**< 0.001**	−0.011 (− 0.028, 0.006)	0.214	−0.003 (− 0.009, 0.003)	0.315
Adjusted model 2^**d, f**^	**−1.62 (− 1.98, − 1.26)**	**< 0.001**	−0.015 (− 0.034, 0.003)	0.112	−0.0006 (− 0.007, 0.006)	0.861
*Girls*						
*Ln waist circumference* ^*b*^						
Adjusted model 1^**c**^	**−0.753 (− 0.809, − 0.697)**	**< 0.001**	**−0.007 (− 0.011, − 0.003)**	**< 0.001**	−0.001 (− 0.002, 0.0005)	0.269
Adjusted model 2^d^	**−0.751 (− 0.813, − 0.688)**	**< 0.001**	**−0.006 (− 0.011, − 0.002)**	**0.003**	−0.001 (− 0.002, 0.0005)	0.260
*Systolic blood pressure*						
Adjusted model 1^**c, e**^	**−16.4 (− 21.4, − 11.4)**	**< 0.001**	−0.1 (− 0.4, 0.2)	0.595	−0.02 (− 0.11, 0.06)	0.624
Adjusted model 2^**d, e**^	**−17.9 (− 23.4, − 12.4)**	**< 0.001**	−0.1 (− 0.4, 0.2)	0.562	−0.005 (− 0.10, 0.09)	0.910
*Ln triglyceride* ^*b*^						
Adjusted model 1^**c**^	**−0.568 (− 0.829, − 0.306)**	**< 0.001**	0.007 (− 0.007, 0.022)	0.315	0.001 (− 0.003, 0.006)	0.576
Adjusted model 2^**d**^	**−0.609 (− 0.892, − 0.325)**	**< 0.001**	0.006 (− 0.009, 0.023)	0.422	0.001 (− 0.003, 0.006)	0.561
*Total cholesterol*						
Adjusted model 1^**c**^	**−0.66 (− 1.13, − 0.20)**	**0.005**	−0.02 (− 0.05, 0.0002)	0.052	0.0004 (− 0.007, 0.008)	0.912
Adjusted model 2^**d**^	**−0.72 (− 1.23, − 0.21)**	**0.006**	−0.02 (− 0.05, 0.005)	0.111	−0.001 (− 0.009, 0.007)	0.799
*Low-density lipoprotein*						
Adjusted model 1^**c**^	**−0.68 (− 1.09, − 0.28)**	**< 0.001**	**−0.03 (− 0.05, − 0.004)**	**0.023**	−0.001 (− 0.008, 0.006)	0.782
Adjusted model 2^d^	**−0.71 (− 1.16, − 0.27)**	**0.002**	**−0.02 (− 0.05, − 0.0002)**	**0.048**	−0.002 (− 0.009, 0.005)	0.567
*High-density lipoprotein*						
Adjusted model 1^**c**^	**0.37 (0.22, 0.51)**	**< 0.001**	0.0006 (− 0.008, 0.009)	0.893	0.001 (− 0.001, 0.004)	0.213
Adjusted model 2^**d**^	**0.33 (0.16, 0.49)**	**< 0.001**	0.001 (− 0.008, 0.01)	0.813	0.001 (− 0.001, 0.004)	0.269
*Ln HOMA-IR* ^*b*^						
Adjusted model 1^**c, f**^	**−1.42 (− 1.88, − 0.96)**	**< 0.001**	−0.004 (− 0.030, 0.022)	0.777	0.001 (− 0.007, 0.009)	0.745
Adjusted model 2^**d, f**^	**−1.54 (− 2.04, − 1.03)**	**< 0.001**	−0.009 (− 0.038, 0.020)	0.537	0.003 (− 0.005, 0.012)	0.451
*Boys*						
*Ln waist circumference* ^*b*^						
Adjusted model 1^**c**^	**−0.453 (− 0.507, − 0.399)**	**< 0.001**	−0.001 (− 0.003, 0.002)	0.687	**−0.001 (− 0.002, − 0.0003)**	**0.013**
Adjusted model 2^**d**^	**−0.485 (− 0.545, − 0.425)**	**< 0.001**	−0.002 (− 0.005, 0.002)	0.335	**−0.001 (− 0.003, − 0.0003)**	**0.014**
*Systolic blood pressure*						
Adjusted model 1^**c, e**^	−3.8 (− 8.8, 1.1)	0.131	−0.1 (− 0.3, 0.1)	0.356	−0.06 (− 0.15, 0.04)	0.233
Adjusted model 2^**d, e**^	−3.6 (− 9.1, 1.9)	0.201	−0.1 (− 0.4, 0.1)	0.409	−0.05 (− 0.15, 0.05)	0.356
*Ln triglyceride* ^*b*^						
Adjusted model 1^**c**^	**−0.611 (− 0.859, − 0.363)**	**< 0.001**	**−0.019 (− 0.031, − 0.006)**	**0.003**	−0.003 (− 0.008, 0.001)	0.164
Adjusted model 2^d^	**−0.604 (− 0.877, − 0.330)**	**< 0.001**	**−0.023 (− 0.037, − 0.009)**	**< 0.001**	−0.003 (− 0.008, 0.002)	0.307
*Total cholesterol*						
Adjusted model 1^**c**^	**−0.54 (− 0.90, − 0.18)**	**0.003**	**−0.03 (− 0.05, − 0.01)**	**< 0.001**	−0.002 (− 0.008, 0.005)	0.625
Adjusted model 2^**d**^	**−0.55 (− 0.95, − 0.15)**	**0.007**	**−0.03 (− 0.05, − 0.01)**	**0.003**	−0.002 (− 0.010, 0.005)	0.497
*Low-density lipoprotein*						
Adjusted model 1^**c**^	**−0.40 (− 0.70, − 0.10)**	**0.008**	**−0.02 (− 0.03, − 0.005)**	**0.010**	−0.0005 (− 0.006, 0.005)	0.848
Adjusted model 2^**d**^	**−0.43 (− 0.77, − 0.09)**	**0.012**	**−0.02 (− 0.04, − 0.003)**	**0.017**	−0.001 (− 0.007, 0.005)	0.750
*High-density lipoprotein*						
Adjusted model 1^**c**^	**0.20 (0.06, 0.33)**	**0.003**	0.001 (− 0.006, 0.01)	0.840	0.001 (− 0.001, 0.004)	0.271
Adjusted model 2^**d**^	**0.22 (0.08, 0.37)**	**0.003**	0.003 (− 0.004, 0.01)	0.355	0.001 (− 0.002, 0.003)	0.568
*Ln HOMA-IR* ^*b*^						
Adjusted model 1^**c, f**^	**−1.68 (− 2.15, − 1.20)**	**< 0.001**	−0.014 (− 0.038, 0.009)	0.241	−0.007 (− 0.016, 0.001)	0.097
Adjusted model 2^**d, f**^	**−1.73 (− 2.24, − 1.22)**	**< 0.001**	−0.019 (− 0.044, 0.006)	0.142	−0.005 (− 0.015, 0.004)	0.279

*Abbreviations* min, minutes; CI, confidence interval; HOMA-IR, insulin resistance from Homeostasis Model Assessment. β represents unstandardized regression coefficient

^**a**^Derived by dividing handgrip strength (kg) by body weight (kg)

^**b**^Ln represents natural-log transformed outcome variables where the base of the log was 2.71828

^**c**^Adjusted for gender (when not stratified by gender), household wealth, and maternal education

^**d**^Additionally adjusted for sedentary time, time engaged in moderate-to-vigorous physical activity and awake wear time (all in minutes per day)

^**e**^The models for association of standing long jump with systolic blood pressure were also adjusted for height (cm)

^**f**^Additionally adjusted for sample storage time as it showed a negative correlation with fasting insulin level (Spearman’s ρ = −0.2, *P* < 0.001)

Statistically significant coefficients are presented in bold

## Discussion

We explored cross-sectional associations of objectively measured PA and fitness with conventional cardiometabolic risk indicators in a rural birth cohort from Bangladesh. Higher total PA was associated with lower WC and IR, but the differences between the top and bottom tertiles were small. There was also a gender-specific, negative association of total PA with SBP among the boys. ST was positively associated with WC and IR, the latter being more pronounced among boys than girls. Although time spent in MVPA and VPA both displayed negative associations with WC, SBP and IR, and positive associations with HDL; the associations were notably stronger for VPA. Greater time spent in VPA, but not in MVPA, was associated with lower TG. Isotemporal substitution models corroborated the stronger impact of VPA than MVPA, as replacement of ST with equal volume of VPA, but not MPA, resulted in lower WC, SBP, IR and TG. The associations of VPA and MVPA with the indicators– except WC– were generally more pronounced among the boys than girls. We found beneficial associations of upper body fitness with all the indicators with large unstandardized effect sizes. Among the boys, however, neither grip strength nor standing long jump showed any association with SBP. Better lower body and cardiorespiratory fitness was associated with a small reduction in WC, but not IR.

The small differences in central adiposity and insulin resistance by total PA levels observed in our study are in line with previous studies among adolescents in North America, Europe and Oceania [52]. Deriving total PA from accelerometry data does not require application of cut-points, and thus, avoids the caveats [53] of defining PA intensities based on predefined cut-points. Nonetheless, total PA does not indicate the relative contribution of PA of different intensities to the accumulated PA. Whereas total PA at any intensity appears to be negatively associated with cardiometabolic risk indicators among middle-aged and older adults [54–56], it is the share of PA of higher intensity that drives these associations among adolescents [57, 58]. The reason for the gender-specific association between higher total PA and lower SBP among the boys remains unclear.

The weak, positive associations of ST with central adiposity and IR in this study support similar findings from recent studies [57, 58]. Conversely, several studies from high-income settings did not demonstrate any association between ST and cardiometabolic indicators including WC and IR among adolescents [59, 60]. This disagreement could be related partly to the use of longer epochs and consequent under-estimation of ST in those reporting no association [31]; and partly to differences in sedentary bout duration [61] and how the ST was spent (sitting and reclined versus otherwise) [57] across the studies. However, epidemiological studies suggest that the association of ST with cardiometabolic risk strengthens substantially in adulthood [56, 62]. The association with WC was notable only among the girls in our study. We speculate this to be an indication of gender-based differences in accumulation of ST in longer bouts and in sitting. For instance, the median ST accumulated in bouts of ≥ 10 min per day was higher among the girls than boys (333 versus 323 min, data not shown). Further studies are needed to explore the variation in behavioral patterning of ST and its accumulation by gender among LMIC adolescents.

In agreement with existing literature [57, 58, 60], our findings delineate the healthful impact of engaging in MVPA and VPA on the cardiometabolic indicators– apart from TC and LDL. The underlying mechanisms are considered multi-dimensional– ranging from functional adaptations and structural remodelling of the cardiovascular system [63] to regulation of adipose tissue function and key enzymes like lipoprotein lipase [64]. The direct comparability of the associations with those in other studies is limited by methodological and analytical differences regarding accelerometer placement, epoch length and cut-points used [31, 53]. Except for WC, the associations were generally prominent among the boys than the girls. This may have resulted from the lower engagement of the girls in VPA– the component in the PA intensity spectrum (in CPM) that imparts the strongest influence on cardiometabolic risk indicators among adolescents [57, 58]. Contrastingly, greater time spent in PA at the lower end of the intensity spectrum has been found to be able to lower WC [65]. Of note, the median percentages of daily wear time spent in VPA were 0.3% among the boys and 0.05% among the girls (Table 1). Consistent with a series of recent studies from high-income countries [57, 58, 66, 67], the magnitude of associations in our analyses was higher for VPA than MVPA or moderate PA. Summarizing PA over longer epochs (e.g., 60 s) tends to misclassify or mask sporadic, intermittent bursts of high-intensity PA [68] which are common among adolescents [32]. As we opted for a shorter, five-second epoch, our analyses avoided this; and thus, the considerably large unstandardized effect sizes for each 10 min of VPA were unsurprising. This alongside the findings from isotemporal substitution models lends support to the emerging notion that the role of VPA is crucial for cardiometabolic health among older children and adolescents who seem to achieve the health benefits during intermittent PA of vigorous intensity [58]. The strength of associations observed for VPA appears meaningful from a public health perspective and support a re-orientation of focus on VPA as a modifiable target for improving cardiometabolic health among adolescents in resource-limited settings.

We found that higher weight-normalized grip strength was associated with lower central adiposity, SBP, IR, and a favorable fasting lipid profile. The associations are consistent with those reported in the literature on physical fitness among adolescents [20, 39]. The magnitude of the associations was substantial and similar to that observed in studies that normalized handgrip strength for body mass [39]. The reported associations of handgrip strength with common cardiometabolic indicators appear to be stronger in studies using weight-normalized grip strength than those using the absolute values for analysis [39]. Standing long jump was negatively associated with WC among the girls and TG among the boys. The reason for such gender specificity was not clear. Contrasting with previous studies from other countries [69, 70], muscular fitness was not associated with SBP among the boys. The lack of association with standing long jump test was particularly unanticipated as common VPA– running or short sprinting, cycling– involve lower extremity muscles more [71], and boys engaged in more VPA than girls in this study. The reason for this remains unclear, but could be related to confounding from lean muscle mass, which shows a positive association with SBP among adolescents [72–74]. Furthermore, VO_2max_ was not associated with any indicator except WC, while previous studies linked VO_2max_ with IR [75, 76] and composite cardiometabolic risk score [77] among adolescents. Whether the level of imprecision in VO_2max_ estimation in CST and any deviation from the prompted stepping rate and rhythm [29] played a role in apparent non-association needs to be investigated.

Given the present study involved rural adolescents, whether the associations of PA and fitness with cardiometabolic risk would differ among urban adolescents is a pertinent question. In a multi-country sample (*n* = 4852; mean age 14.6 years) that included 90 urban adolescents from Dhaka, Van Dyck et al. [78] found a 10 min/day higher MVPA was associated with 0.04 SD lower BMI. As the study did not examine associations with any other cardiometabolic indicator, the reported findings are not directly comparable to ours. Several studies suggest built-environment factors including space available for outdoor sports, pedestrian and cycling infrastructure, and mode of active commuting to school may drive differences in the share of VPA in habitual movement behavior among urban versus rural adolescents [79–82]. Further research is warranted to pinpoint whether these translate into urban-rural differences in the direction or magnitude of associations of PA and fitness with cardiometabolic risk markers.

Key strengths of the study include: a moderately large sample size from a well-characterized birth cohort [26]; field-based application of reliable, valid methods for objective assessment of PA and fitness in a low-income setting; and adherence to standardized protocol for data collection and analytical criteria for PA (≥ 10 h observation per day and ≥ 5 valid days including one weekend day) for representing habitual PA. Accelerometers produce reasonably accurate PA data from general populations in free-living conditions [83]. The cut-points in this study were developed from ActiGraph accelerometers placed in non-dominant wrist using five-second epoch [36]. We used the same accelerometer placement and identical epoch length in our analysis to ensure reliable estimates [31]. Hence, the estimates of associations are expected to be valid and actionable from a public health perspective. The findings are generalizable to adolescents in Matlab because of the area-wide recruitment of pregnant women in the MINIMat trial [25] and also to other rural settings in Bangladesh due to similarity in socio-cultural context. The limitations concerning accelerometry-related issues deserve a thorough consideration. Although wrist-mounted accelerometers increase wear compliance [31], wrist placement appears less sensitive than thigh placement for stationary activities, cycling and some sitting postures associated with varied wrist positions [84]. Use of activity count-based cut-points appears to generate higher estimates of MVPA compared to gravitational unit-based cut-offs (Euclidean norm minus one, ENMO) [53]– a potential source of misclassification in our study. Non-wear time exclusion is another classification-misclassification trade-off as it is not possible to distinguish ST or sleep from non-wear time with a 100% accuracy [85]. We chose an algorithm [34] that allows non-zero interruptions; so that abrupt touching or wrist movement and spurious spikes during a non-wear period do not convert the non-wear period into sedentary time [86]. However, there is evidence that algorithms allowing interruptions may influence classification accuracy [85]. To keep wearing accelerometer over a period can be obtrusive and wearing an accelerometer itself has been shown to increase PA [87]. Drawing any causal inference from the associations observed would be erroneous owing to the cross-sectional design. Finally, we could not completely rule out residual confounding.

## Conclusion

Higher PA and muscular fitness were associated with a healthier cardiometabolic profile in terms of WC, SBP, IR and some lipid markers among the MINIMat adolescents. The associations were markedly strong for VPA and upper body muscular fitness, and generally more pronounced among the boys. For a better understanding of the gender-based differences, future studies need to explore types of PA and how ST and intensity-specific PA accumulate in terms of number and duration of bouts. The findings highlighted VPA and muscular fitness as modifiable correlates of interest for preventive strategies targeting adolescents in a rural, LMIC setting.

## Electronic Supplementary Material

Below is the link to the electronic supplementary material.

Supplementary Material 1

## Data Availability

The present study utilized data from the 15-year follow-up of the MINIMat (Maternal and Infant Nutrition Interventions in Matlab) trial. The 15-year follow-up was a large, collaborative project involving Uppsala University, Karolinska Institute, Finnish Institute for Health and Welfare, University of Oulu and International Centre for Diarrhoeal Disease Research, Bangladesh (icddr, b). Because of the statutory requirements, internal data policies and regulations existing in the collaborating bodies along with the over-arching General Data Protection Regulation (GDPR), the data must be stored in institutional repository (storage platforms) and cannot be made directly accessible without a review of the request for access to data. Data availability is further limited because the data contain information on gender and health-related and behavioral attributes, and thus, considered to be “sensitive personal data” as per GDPR. While the data are pseudonymized in accordance with GDPR, attributes that can link the data to each study participant exist and are preserved following regulations in place at the collaborating bodies. Therefore, the data can be accessed only upon a formal request that details the purpose of such request. Requests to access these datasets should be directed to: E-CE (email: lotta. ekstrom@kbh.uu.se).

## Abbreviations

BMI: Body mass index
CST: Chester Step Test
CI: Confidence interval
HDL: High-density lipoprotein
HOMA-IR: Homeostasis Model Assessment-insulin resistance
icddr,b: International Centre for Diarrhoeal Disease Research, Bangladesh
LDL: Low-density lipoprotein
LIPA: Light-intensity physical activity
LMIC: Low- and middle-income country
MINIMat: Maternal and Infant Nutrition Interventions in Matlab
MVPA: Moderate-to-vigorous physical activity
PA: Physical activity
ST: Sedentary time
SBP: Systolic blood pressure
TC: Total cholesterol
TG: Triglyceride
VIF: Variance inflation factor
VPA: Vigorous physical activity
WC: Waist circumference
