# Supplementary material for: Accelerometer-Measured Physical Activity, Fitness and Indicators of Cardiometabolic Risk among Rural Adolescents: A Cross-Sectional Study at 15-Year Follow-up of the MINIMat Cohort
Source: J Epidemiol Glob Health. 2024 May 21;14(3):987–1003. doi: 10.1007/s44197-024-00245-1 (PMC11442897; doi:10.1007/s44197-024-00245-1)
Supplement: Supplementary file 1 — Supplementary Material 1 [file 44197_2024_245_MOESM1_ESM.docx]

**Supplementary Materials**

**Accelerometer-measured physical activity, fitness and indicators of cardiometabolic risk among rural adolescents: a cross-sectional study at 15-year follow-up of the MINIMat cohort**

Mohammad Redwanul Islam**^1^**, Christine Delisle Nyström**^2^**, Maria Kippler**^3^**, Eero Kajantie**^4,5^**, Marie Löf**^2,6^**, Syed Moshfiqur Rahman**^1,7^**, Eva-Charlotte Ekström**^1^**

^1^Department of Women’s and Children’s Health, Uppsala University, Uppsala, Sweden, ^2^Department of Biosciences and Nutrition, Karolinska Institutet, Huddinge, Sweden, ^3^Institute of Environmental Medicine, Unit of Metals and Health, Karolinska Institutet, Stockholm, Sweden, ^4^Department of Public Health and Welfare, Finnish Institute for Health and Welfare, Helsinki, Finland, ^5^PEDEGO Research Unit, MRC Oulu, Oulu University Hospital & University of Oulu, Oulu, Finland, ^6^Department of Health, Medicine and Caring Sciences, Linköping University, Linköping, Sweden, ^7^Maternal and Child Health Division, International Centre for Diarrhoeal Disease Research, Bangladesh (icddr,b), Dhaka, Bangladesh

**Corresponding author:** Mohammad Redwanul Islam; mohammadredwanul.islam@kbh.uu.se

**
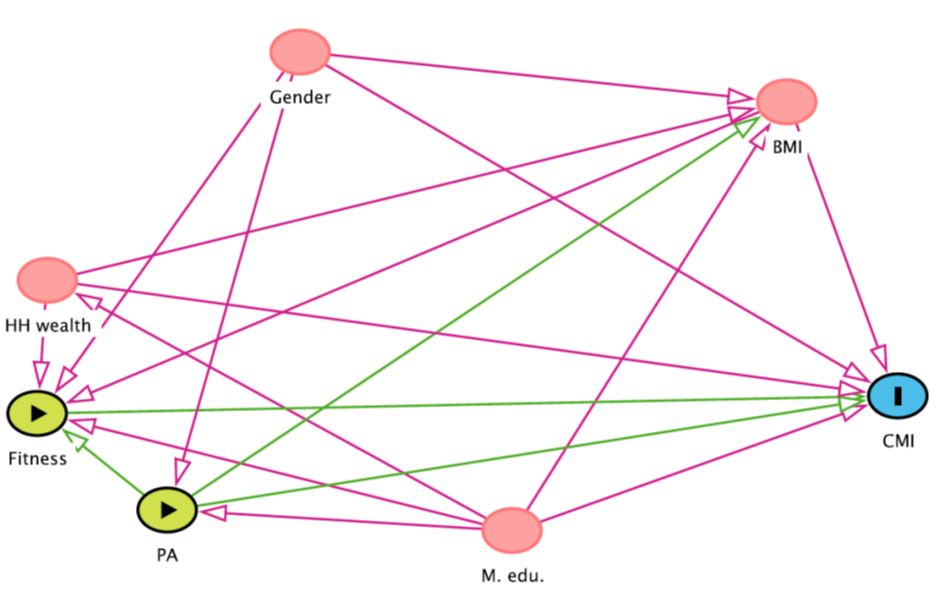
**

**Supplementary Figure 1.** Directed acyclic graph for the relationship of physical activity and fitness with indicators of cardiometabolic risk prepared in dagitty.net. The pink arrows indicate biasing paths and the green arrows indicate causal paths. Green circles indicate the main exposures (physical activity and fitness) and the blue one indicates the outcome. PA, physical activity; HH, household; M. edu., maternal education; BMI, body mass index; CMI, cardiometabolic indicators.

**Supplementary Table 1.** Unstandardized regression coefficients from isotemporal substitution models indicating the impact of replacing each 10 minutes/day of sedentary time with 10 minutes/day of intensity-specific physical activity among the adolescents

| **Indicator** | **LIPA^1^** | | **MPA^1^** | | **VPA^1^** | |
| --- | --- | --- | --- | --- | --- | --- |
|  | β (95% CI) | P-value | β (95% CI) | P-value | β (95% CI) | P-value |
| Ln waist circumference**^2^** | −0.001  (−0.002, 0.001) | 0.438 | −0.001  (−0.004, 0.003) | 0.722 | **−0.047**  **(−0.072, −0.022)** | **<0.001** |
| Systolic blood pressure | −0.06  (−0.19, 0.07) | 0.389 | 0.06  (−0.19, 0.32) | 0.615 | **−3.33**  **(−5.26, −1.40)** | **<0.001** |
| Ln triglyceride**^2^** | −0.005  (−0.011, 0.002) | 0.164 | 0.007  (−0.005, 0.02) | 0.245 | **−0.14**  **(−0.23, −0.04)** | **0.006** |
| Total cholesterol | −0.003  (−0.01, 0.007) | 0.524 | 0.004  (−0.02, 0.02) | 0.710 | 0.01  (−0.15, 0.16) | 0.939 |
| Low-density lipoprotein | −0.005  (−0.01, 0.004) | 0.265 | 0.01  (−0.01, 0.02) | 0.381 | 0.04  (−0.18, 0.09) | 0.548 |
| High-density lipoprotein | 0.002  (−0.001, 0.006) | 0.262 | −0.002  (−0.01, 0.005) | 0.621 | **0.11**  **(0.05, 0.16)** | **<0.001** |
| Ln HOMA-IR**^2,3^** | **−0.014**  **(−0.027, −0.002)** | **0.021** | 0.012  (−0.011, 0.035) | 0.309 | **−0.30**  **(−0.476, −0.119)** | **0.001** |

Abbreviations: LIPA, light-intensity physical activity; MPA, moderate physical activity; VPA, vigorous physical activity; CI, confidence interval; HOMA-IR, insulin resistance from Homeostasis Model Assessment. β represents unstandardized regression coefficient. **^1^**Model with variables: LIPA (minutes/day), MPA (minutes/day), VPA (minutes/day), awake wear time (minutes/day), gender, household wealth, and maternal education, in accordance with Mekary et al. (Reference number 49). **^2^**Ln represents natural-log transformed variables where the base of the log was 2.71828. **^3^**Additionally adjusted for sample storage time as it showed a negative correlation with fasting insulin level (Spearman’s ρ = –0.2, P<0.001). Statistically significant coefficients are presented in bold.

**Supplementary Table 2.** Values of variance inflation factor for the variables included in the isotemporal substitution models

| **Variables in the isotemporal model** | **Variance inflation factor**  **(95% confidence interval)** |
| --- | --- |
| Light-intensity physical activity (minutes/day) | 2.45 (2.28, 2.63) |
| Moderate physical activity (minutes/day) | 2.54 (2.36, 2.73) |
| Vigorous physical activity (minutes/day) | 1.89 (1.77, 2.02) |
| Awake wear time (minutes/day) | 1.30 (1.23, 1.38) |
| Gender^1^ | 1.72 (1.62, 1.84) |
| Household wealth^1^ | 1.24 (1.18, 1.32) |
| Maternal education**^1^** | 1.22 (1.16, 1.30) |

**^1^**For these categorical variables, the values represent generalized variance inflation factor (GVIF) and the corresponding values for GVIF raised to the power of 1/(2×degrees of freedom) were: 1.31 (Gender), 1.05 (household wealth), and 1.05 (for maternal education). The values of GVIF^1/(2×degrees of freedom)^ squared are equivalent to and interpretable in the same way as a VIF.

**Table 3.** Associations of time spent sedentary and in moderate, vigorous and moderate-to-vigorous physical activity with the selected cardiometabolic indicators per 10-minute change.

| **Indicator** | **Time spent sedentary**  (minutes/day) | | **Time spent in MPA**  (minutes/day) | | **Time spent in VPA**  (minutes/day) | | **Time spent in MVPA**  (minutes/day) | |
| --- | --- | --- | --- | --- | --- | --- | --- | --- |
|  | β (95% CI) | P-value | β (95% CI) | P-value | β (95% CI) | P-value | β (95% CI) | P-value |
| **Entire sample** | | | | | | | | |
| **Ln waist circumference^1^** |  |  |  |  |  |  |  |  |
| Unadjusted | **0.0005 (0.00001, 0.001)** | **0.045** | **−0.005 (−0.007, −0.002)** | **<0.001** | **−0.04 (−0.06, −0.02)** | **<0.001** | **−0.005 (−0.007, −0.003)** | **<0.001** |
| Adjusted**^2^** | **0.001 (0.0004, 0.002)** | **0.005** | **−0.003 (−0.006, −0.001)** | **0.001** | **−0.05 (−0.07, −0.03)** | **<0.001** | **−0.004 (−0.006, −0.002)** | **<0.001** |
| **Systolic blood pressure** |  |  |  |  |  |  |  |  |
| Unadjusted | 0.01 (−0.03, 0.05) | 0.653 | **−0.22 (−0.38, −0.06)** | **0.007** | 0.52 (−0.91, 1.95) | 0.479 | **−0.20 (−0.35, −0.04)** | **0.012** |
| Adjusted**^2^** | 0.06 (−0.01, 0.12) | 0.085 | −0.16 (−0.32, 0.01) | 0.065 | **−3.17 (−4.82, −1.52)** | **<0.001** | **−0.17 (−0.33, −0.01)** | **0.034** |
| **Ln triglyceride^1^** |  |  |  |  |  |  |  |  |
| Unadjusted | 0.002 (−0.0003, 0.004) | 0.088 | 0.0006 (−0.007, 0.009) | 0.885 | **−0.23 (−0.30, −0.16)** | **<0.001** | −0.002 (−0.01, 0.006) | 0.590 |
| Adjusted**^2^** | 0.002 (−0.001, 0.005) | 0.193 | −0.004 (−0.012, 0.004) | 0.334 | **−0.11 (−0.19, −0.03)** | **0.008** | −0.005 (−0.013, 0.003) | 0.240 |
| **Total cholesterol** |  |  |  |  |  |  |  |  |
| Unadjusted | **0.005 (0.001, 0.008)** | **0.005** | 0.01 (−0.004, 0.022) | 0.197 | **−0.31 (−0.43, −0.19)** | **<0.001** | 0.004 (−0.008, 0.017) | 0.494 |
| Adjusted**^2^** | 0.001 (−0.005, 0.006) | 0.762 | 0.0003 (−0.013, 0.014) | 0.964 | 0.02 (−0.12, 0.15) | 0.815 | 0.0004 (−0.012, 0.013) | 0.948 |
| **Low-density lipoprotein** |  |  |  |  |  |  |  |  |
| Unadjusted | **0.004 (0.001, 0.007)** | **0.006** | 0.006 (−0.005, 0.017) | 0.302 | **−0.27 (−0.37, −0.17)** | **<0.001** | 0.002 (−0.009, 0.013) | 0.677 |
| Adjusted**^2^** | 0.001 (−0.003, 0.006) | 0.610 | 0.0001 (−0.011, 0.012) | 0.983 | −0.02 (−0.13, 0.10) | 0.773 | −0.00004 (−0.011, 0.011) | 0.994 |
| **High-density lipoprotein** |  |  |  |  |  |  |  |  |
| Unadjusted | −0.0005 (−0.001, 0.001) | 0.384 | **0.006 (0.001, 0.01)** | **0.009** | **0.09 (0.05, 0.13)** | **<0.001** | **0.006 (0.002, 0.011)** | **0.003** |
| Adjusted**^2^** | **−0.002 (−0.004, −0.0003)** | **0.024** | **0.006 (0.001, 0.01)** | **0.018** | **0.10 (0.06, 0.15)** | **<0.001** | **0.006 (0.002, 0.01)** | **0.007** |
| **Ln HOMA-IR^1^** |  |  |  |  |  |  |  |  |
| Unadjusted | **0.005 (0.001, 0.009)** | **0.006** | −0.015 (−0.03, 0.0005) | 0.057 | **−0.45 (−0.58, −0.31)** | **<0.001** | **−0.019 (−0.033, −0.004)** | **0.012** |
| Adjusted**^2, 3^** | **0.009 (0.003, 0.015)** | **0.004** | **−0.018 (−0.033, −0.002)** | **0.023** | **−0.28 (−0.43, −0.12)** | **<0.001** | **−0.019 (−0.033, −0.004)** | **0.012** |
| **Girls** | | | | | | | | |
| **Ln waist circumference^1^** |  |  |  |  |  |  |  |  |
| Unadjusted | 0.0004 (−0.0004, 0.001) | 0.330 | **−0.005 (−0.008, −0.002)** | **0.002** | **−0.10 (−0.19, −0.02)** | **0.014** | **−0.005 (−0.008, −0.002)** | **0.002** |
| Adjusted**^2^** | **0.001 (0.0002, 0.003)** | **0.023** | **−0.004**  **(−0.007, −0.0005)** | **0.023** | **−0.09 (−0.17, −0.01)** | **0.035** | **−0.004**  **(−0.007, −0.0005)** | **0.021** |
| **Systolic blood pressure** |  |  |  |  |  |  |  |  |
| Unadjusted | 0.001 (−0.05, 0.05) | 0.964 | 0.02 (−0.20, 0.23) | 0.872 | −2.55 (−8.43, 3.32) | 0.394 | 0.01 (−0.20, 0.22) | 0.898 |
| Adjusted**^2^** | −0.05 (−0.14, 0.04) | 0.296 | 0.01 (−0.21, 0.23) | 0.917 | −2.32 (−8.21, 3.58) | 0.440 | 0.01 (−0.21, 0.23) | 0.942 |
| **Ln triglyceride^1^** |  |  |  |  |  |  |  |  |
| Unadjusted | 0.000004 (−0.003, 0.003) | 0.998 | 0.002 (−0.008, 0.01) | 0.646 | 0.13 (−0.17, 0.43) | 0.386 | 0.003 (−0.008, 0.013) | 0.629 |
| Adjusted**^2^** | 0.0004 (−0.004, 0.005) | 0.875 | −0.00002  (−0.011, 0.011) | 0.997 | 0.10 (−0.19, 0.40) | 0.497 | 0.0001 (−0.011, 0.011) | 0.983 |
| **Total cholesterol** |  |  |  |  |  |  |  |  |
| Unadjusted | 0.004 (−0.001, 0.008) | 0.13 | 0.0003 (−0.019, 0.02) | 0.974 | −0.09 (−0.62, 0.45) | 0.747 | 0.0002 (−0.019, 0.019) | 0.984 |
| Adjusted**^2^** | 0.002 (−0.006, 0.010) | 0.633 | −0.004 (−0.024, 0.017) | 0.721 | −0.09 (−0.62, 0.45) | 0.748 | −0.004 (−0.024, 0.016) | 0.717 |
| **Low-density lipoprotein** |  |  |  |  |  |  |  |  |
| Unadjusted | 0.002 (−0.002, 0.007) | 0.244 | 0.004 (−0.013, 0.021) | 0.632 | −0.16 (−0.62, 0.31) | 0.508 | 0.004 (−0.013, 0.02) | 0.656 |
| Adjusted**^2^** | 0.0001 (−0.007, 0.007) | 0.985 | 0.002 (−0.016, 0.02) | 0.820 | −0.15 (−0.61, 0.32) | 0.531 | 0.002 (−0.016, 0.019) | 0.843 |
| **High-density lipoprotein** |  |  |  |  |  |  |  |  |
| Unadjusted | 0.001 (−0.001, 0.002) | 0.329 | −0.002 (−0.008, 0.005) | 0.591 | 0.02 (−0.15, 0.19) | 0.8 | −0.002 (−0.008, 0.004) | 0.604 |
| Adjusted**^2^** | 0.0005 (−0.002, 0.003) | 0.691 | −0.002 (−0.009, 0.004) | 0.532 | 0.03 (−0.14, 0.20) | 0.736 | −0.002 (−0.01, 0.004) | 0.548 |
| **Ln HOMA-IR^1^** |  |  |  |  |  |  |  |  |
| Unadjusted | 0.001 (−0.004, 0.006) | 0.642 | −0.017 (−0.038, 0.003) | 0.096 | −0.28 (−0.83, 0.28) | 0.33 | −0.017 (−0.037, 0.003) | 0.095 |
| Adjusted**^2,3^** | 0.005 (−0.003, 0.001) | 0.215 | −0.01 (−0.03, 0.001) | 0.310 | −0.19 (−0.73, 0.35) | 0.492 | −0.01 (−0.03, 0.01) | 0.307 |
| **Boys** | | | | | | | | |
| **Ln waist circumference^1^** |  |  |  |  |  |  |  |  |
| Unadjusted | 0.001 (−0.000004, 0.001) | 0.051 | **−0.004 (−0.007, −0.001)** | **0.005** | **−0.05 (−0.08, −0.03)** | **<0.001** | **−0.004 (−0.007, −0.002)** | **0.001** |
| Adjusted**^2^** | 0.001 (−0.0002, 0.002) | 0.092 | **−0.003**  **(−0.007, −0.0005)** | **0.024** | **−0.05 (−0.07, −0.02)** | **<0.001** | **−0.004 (−0.007, −0.001)** | **0.009** |
| **Systolic blood pressure** |  |  |  |  |  |  |  |  |
| Unadjusted | 0.04 (−0.02, 0.10) | 0.213 | **−0.37 (−0.61, −0.14)** | **0.002** | **−3.37 (−5.14, −1.60)** | **<0.001** | **−0.37 (−0.59, −0.15)** | **<0.001** |
| Adjusted**^2^** | **0.15 (0.05, 0.25)** | **0.002** | **−0.32 (−0.57, −0.07)** | **0.012** | **−3.08 (−4.87, −1.29)** | **<0.001** | **−0.32 (−0.55, −0.09)** | **0.006** |
| **Ln triglyceride^1^** |  |  |  |  |  |  |  |  |
| Unadjusted | 0.002 (−0.0003, 0.005) | 0.083 | −0.006 (−0.018, 0.005) | 0.274 | **−0.12 (−0.21, −0.03)** | **0.006** | −0.01 (−0.02, 0.003) | 0.087 |
| Adjusted**^2^** | 0.004 (−0.001, 0.008) | 0.121 | −0.01 (−0.02, 0.004) | 0.184 | **−0.12 (−0.21, −0.03)** | **0.007** | −0.01 (−0.02, 0.002) | 0.117 |
| **Total cholesterol** |  |  |  |  |  |  |  |  |
| Unadjusted | 0.004 (−0.0005, 0.008) | 0.081 | 0.007 (−0.01, 0.02) | 0.421 | 0.02 (−0.10, 0.15) | 0.7 | 0.006 (−0.01, 0.022) | 0.428 |
| Adjusted**^2^** | −0.0003 (−0.007, 0.007) | 0.922 | 0.004 (−0.014, 0.022) | 0.631 | 0.04 (−0.09, 0.17) | 0.586 | 0.004 (−0.012, 0.02) | 0.608 |
| **Low-density lipoprotein** |  |  |  |  |  |  |  |  |
| Unadjusted | **0.004 (0.0003, 0.007)** | **0.033** | −0.0005 (−0.015, 0.014) | 0.943 | −0.01 (−0.12, 0.10) | 0.859 | −0.001 (−0.014, 0.013) | 0.929 |
| Adjusted**^2^** | 0.002 (−0.004, 0.008) | 0.458 | −0.002 (−0.017, 0.013) | 0.791 | 0.004 (−0.10, 0.11) | 0.945 | −0.002 (−0.016, 0.012) | 0.814 |
| **High-density lipoprotein** |  |  |  |  |  |  |  |  |
| Unadjusted | **−0.002 (−0.003,−0.0002)** | **0.028** | **0.014 (0.008, 0.02)** | **<0.001** | **0.11 (0.07, 0.16)** | **<0.001** | **0.014 (0.008, 0.020)** | **<0.001** |
| Adjusted**^2^** | **−0.004 (−0.007,−0.002)** | **<0.001** | **0.014 (0.007, 0.02)** | **<0.001** | **0.11 (0.06, 0.15)** | **<0.001** | **0.013 (0.007, 0.02)** | **<0.001** |
| **Ln HOMA-IR^1^** |  |  |  |  |  |  |  |  |
| Unadjusted | **0.008 (0.002, 0.013)** | **0.021** | −0.022 (−0.044, 0.001) | 0.061 | **−0.25 (−0.42, −0.08)** | **0.004** | **−0.022 (−0.043, −0.001)** | **0.037** |
| Adjusted**^2,3^** | **0.013 (0.004, 0.022)** | **0.006** | **−0.02 (−0.05, −0.002)** | **0.03** | **−0.30 (−0.47, −0.13)** | **<0.001** | **−0.027 (−0.048, −0.005)** | **0.015** |

Abbreviations: MPA, moderate physical activity; VPA, vigorous physical activity; MVPA, moderate-to-vigorous physical activity; CI, confidence interval; HOMA-IR, insulin resistance from Homeostasis Model Assessment. β represents unstandardized regression coefficient. 1Ln represents natural-log transformed outcome variables where the base of the log was 2.71828. 2Adjusted for gender (when not stratified by gender), household wealth, maternal education and awake wear time (minutes/day). 3Additionally adjusted for sample storage time as it showed a negative correlation with fasting insulin level (Spearman’s ρ = –0.2, P<0.001). Statistically significant coefficients are presented in bold.

**Table 4.** Associations of weight-normalized handgrip strength, standing long jump (per 10-centimeter increase) and maximal oxygen consumption with the selected cardiometabolic indicators

| **Indicator** | **Weight-normalized grip strength^a^** | | **Standing long jump (cm)** | | **Maximal oxygen consumption**  **(mL/kg/min)** | |
| --- | --- | --- | --- | --- | --- | --- |
|  | β (95% CI) | P-value | β (95% CI) | P-value | β (95% CI) | P-value |
| **Entire sample** | | | | | | |
| **Ln waist circumference^b^** |  |  |  |  |  |  |
| Unadjusted model | **−0.373 (−0.406, −0.340)** | **<0.001** | −0.002 (−0.003, 0.00002) | 0.053 | **−0.001 (−0.002, −0.0004)** | **0.004** |
| Adjusted model 1**^c^** | **−0.589 (−0.628, −0.550)** | **<0.001** | **−0.003 (−0.005, −0.001)** | **0.009** | **−0.001 (−0.002, −0.0002)** | **0.010** |
| Adjusted model 2**^d^** | **−0.604 (−0.647, −0.560)** | **<0.001** | **−0.003 (−0.006, −0.001)** | **0.011** | **−0.001 (−0.002, −0.0003)** | **0.010** |
| **Systolic blood pressure** |  |  |  |  |  |  |
| Unadjusted model | 1.4 (−1.3, 4.2) | 0.309 | **0.5 (0.4, 0.6)** | **<0.001** | −0.004 (−0.07, 0.06) | 0.889 |
| Adjusted model 1**^c,e^** | **−9.5 (−13.1, −6.0)** | **<0.001** | −0.1 (−0.2, 0.1) | 0.483 | −0.04 (−0.10, 0.02) | 0.206 |
| Adjusted model 2**^d,e^** | **−9.7 (−13.6, −5.8)** | **<0.001** | −0.1 (−0.3, 0.1) | 0.462 | −0.02 (−0.09, 0.04) | 0.508 |
| **Ln triglyceride^b^** |  |  |  |  |  |  |
| Unadjusted model | **−0.738 (−0.977, −0.599)** | **<0.001** | **−0.026 (−0.033, −0.019)** | **<0.001** | −0.003 (−0.006, 0.0003) | 0.074 |
| Adjusted model 1**^c^** | **−0.590 (−0.769, −0.410)** | **<0.001** | −0.008 (−0.017, 0.001) | 0.09 | −0.001 (−0.004, 0.002) | 0.514 |
| Adjusted Model 2**^d^** | **−0.599 (−0.794, −0.403)** | **<0.001** | **−0.011 (−0.021, −0.001)** | **0.034** | −0.001 (−0.004, 0.003) | 0.693 |
| **Total cholesterol** |  |  |  |  |  |  |
| Unadjusted | **−1.25 (−1.47, −1.02)** | **<0.001** | **−0.06 (−0.07, −0.05)** | **<0.001** | −0.005 (−0.010, 0.0003) | 0.066 |
| Adjusted model 1**^c^** | **−0.59 (−0.89, −0.30)** | **<0.001** | **−0.03 (−0.04, −0.01)** | **<0.001** | −0.001 (−0.006, 0.004) | 0.796 |
| Adjusted model 2**^d^** | **−0.63 (−0.95, −0.31)** | **<0.001** | **−0.03 (−0.04, −0.01)** | **0.001** | −0.002 (−0.007, 0.004) | 0.502 |
| **Low-density lipoprotein** |  |  |  |  |  |  |
| Unadjusted model | **−1.05 (−1.24, −0.86)** | **<0.001** | **−0.05 (−0.06, −0.04)** | **<0.001** | −0.004 (−0.009, 0.0001) | 0.058 |
| Adjusted model 1**^c^** | **−0.53 (−0.78, −0.28)** | **<0.001** | **−0.02 (−0.03, −0.009)** | **0.001** | −0.001 (−0.005, 0.003) | 0.718 |
| Adjusted model 2**^d^** | **−0.55 (−0.83, −0.28)** | **<0.001** | **−0.02 (−0.03, −0.007)** | **0.003** | −0.002 (−0.006, 0.003) | 0.497 |
| **High-density lipoprotein** |  |  |  |  |  |  |
| Unadjusted model | **0.18 (0.11, 0.26)** | **<0.001** | 0.001 (−0.002, 0.005) | 0.494 | 0.002 (−0.0001, 0.003) | 0.066 |
| Adjusted model 1**^c^** | **0.27 (0.17, 0.37)** | **<0.001** | 0.0003 (−0.005, 0.005) | 0.907 | 0.001 (−0.0002, 0.003) | 0.094 |
| Adjusted model 2**^d^** | **0.26 (0.15, 0.37)** | **<0.001** | 0.002 (−0.004, 0.008) | 0.504 | 0.001 (−0.0007, 0.003) | 0.198 |
| **Ln HOMA-IR^b^** |  |  |  |  |  |  |
| Unadjusted model | **−1.72 (−1.98, −1.46)** | **<0.001** | **−0.044 (−0.057, −0.031)** | **<0.001** | −0.005 (−0.011, 0.001) | 0.099 |
| Adjusted model 1**^c,f^** | **−1.56 (−1.89, −1.23)** | **<0.001** | −0.011 (−0.028, 0.006) | 0.214 | −0.003 (−0.009, 0.003) | 0.315 |
| Adjusted model 2**^d,f^** | **−1.62 (−1.98, −1.26)** | **<0.001** | −0.015 (−0.034, 0.003) | 0.112 | −0.0006 (−0.007, 0.006) | 0.861 |
| **Girls** | | | | | | |
| **Ln waist circumference^b^** |  |  |  |  |  |  |
| Unadjusted model | **−0.768 (−0.824, −0.713)** | **<0.001** | **−0.009 (−0.012, −0.005)** | **<0.001** | −0.001 (−0.002, 0.0004) | 0.183 |
| Adjusted model 1**^c^** | **−0.753 (−0.809, −0.697)** | **<0.001** | **−0.007 (−0.011, −0.003)** | **<0.001** | −0.001 (−0.002, 0.0005) | 0.269 |
| Adjusted model 2^d^ | **−0.751 (−0.813, −0.688)** | **<0.001** | **−0.006 (−0.011, −0.002)** | **0.003** | −0.001 (−0.002, 0.0005) | 0.260 |
| **Systolic blood pressure** |  |  |  |  |  |  |
| Unadjusted model | **−16.3 (−21.2, −11.5)** | **<0.001** | −0.05 (−0.3, 0.2) | 0.744 | −0.02 (−0.11, 0.06) | 0.584 |
| Adjusted model 1**^c,e^** | **−16.4 (−21.4, −11.4)** | **<0.001** | −0.1 (−0.4, 0.2) | 0.595 | −0.02 (−0.11, 0.06) | 0.624 |
| Adjusted model 2**^d,e^** | **−17.9 (−23.4, −12.4)** | **<0.001** | −0.1 (−0.4, 0.2) | 0.562 | −0.005 (−0.10, 0.09) | 0.910 |
| **Ln triglyceride^b^** |  |  |  |  |  |  |
| Unadjusted model | **−0.450 (−0.709, −0.191)** | **<0.001** | 0.011 (−0.004, 0.025) | 0.149 | 0.001 (−0.003, 0.006) | 0.485 |
| Adjusted model 1**^c^** | **−0.568 (−0.829, −0.306)** | **<0.001** | 0.007 (−0.007, 0.022) | 0.315 | 0.001 (−0.003, 0.006) | 0.576 |
| Adjusted model 2**^d^** | **−0.609 (−0.892, −0.325)** | **<0.001** | 0.006 (−0.009, 0.023) | 0.422 | 0.001 (−0.003, 0.006) | 0.561 |
| **Total cholesterol** |  |  |  |  |  |  |
| Unadjusted model | **−0.66 (−1.11, −0.20)** | **0.005** | −0.02 (−0.05, 0.001) | 0.056 | 0.0005 (−0.007, 0.008) | 0.898 |
| Adjusted model 1**^c^** | **−0.66 (−1.13, −0.20)** | **0.005** | −0.02 (−0.05, 0.0002) | 0.052 | 0.0004 (−0.007, 0.008) | 0.912 |
| Adjusted model 2**^d^** | **−0.72 (−1.23, −0.21)** | **0.006** | −0.02 (−0.05, 0.005) | 0.111 | −0.001 (−0.009, 0.007) | 0.799 |
| **Low-density lipoprotein** |  |  |  |  |  |  |
| Unadjusted model | **−0.70 (−1.10, −0.30)** | **<0.001** | **−0.03 (−0.05, −0.004)** | **0.021** | −0.001 (−0.008, 0.006) | 0.781 |
| Adjusted model 1**^c^** | **−0.68 (−1.09, −0.28)** | **<0.001** | **−0.03 (−0.05, −0.004)** | **0.023** | −0.001 (−0.008, 0.006) | 0.782 |
| Adjusted model 2^d^ | **−0.71 (−1.16, −0.27)** | **0.002** | **−0.02 (−0.05, −0.0002)** | **0.048** | −0.002 (−0.009, 0.005) | 0.567 |
| **High-density lipoprotein** |  |  |  |  |  |  |
| Unadjusted model | **0.33 (0.19, 0.48)** | **<0.001** | −0.0001 (−0.01, 0.01) | 0.975 | 0.001 (−0.001, 0.004) | 0.243 |
| Adjusted model 1**^c^** | **0.37 (0.22, 0.51)** | **<0.001** | 0.0006 (−0.008, 0.009) | 0.893 | 0.001 (−0.001, 0.004) | 0.213 |
| Adjusted model 2**^d^** | **0.33 (0.16, 0.49)** | **<0.001** | 0.001 (−0.008, 0.01) | 0.813 | 0.001 (−0.001, 0.004) | 0.269 |
| **Ln HOMA-IR^b^** |  |  |  |  |  |  |
| Unadjusted model | **−1.61 (−2.08, −1.14)** | **<0.001** | −0.011 (−0.038, 0.015) | 0.406 | 0.004 (−0.004, 0.012) | 0.379 |
| Adjusted model 1**^c,f^** | **−1.42 (−1.88, −0.96)** | **<0.001** | −0.004 (−0.030, 0.022) | 0.777 | 0.001 (−0.007, 0.009) | 0.745 |
| Adjusted model 2**^d,f^** | **−1.54 (−2.04, −1.03)** | **<0.001** | −0.009 (−0.038, 0.020) | 0.537 | 0.003 (−0.005, 0.012) | 0.451 |
| **Boys** | | | | | | |
| **Ln waist circumference^b^** |  |  |  |  |  |  |
| Unadjusted model | **−0.481 (−0.536, −0.426)** | **<0.001** | −0.001 (−0.004, 0.002) | 0.543 | **−0.002 (−0.003, −0.0005)** | **0.004** |
| Adjusted model 1**^c^** | **−0.453 (−0.507, −0.399)** | **<0.001** | −0.001 (−0.003, 0.002) | 0.687 | **−0.001 (−0.002, −0.0003)** | **0.013** |
| Adjusted model 2**^d^** | **−0.485 (−0.545, −0.425)** | **<0.001** | −0.002 (−0.005, 0.002) | 0.335 | **−0.001 (−0.003, −0.0003)** | **0.014** |
| **Systolic blood pressure** |  |  |  |  |  |  |
| Unadjusted model | **−5.0 (−9.9, −0.07)** | **0.047** | **0.3 (0.04, 0.5)** | **0.02** | −0.07 (−0.16, 0.03) | 0.159 |
| Adjusted model 1**^c,e^** | −3.8 (−8.8, 1.1) | 0.131 | −0.1 (−0.3, 0.1) | 0.356 | −0.06 (−0.15, 0.04) | 0.233 |
| Adjusted model 2**^d,e^** | −3.6 (−9.1, 1.9) | 0.201 | −0.1 (−0.4, 0.1) | 0.409 | −0.05 (−0.15, 0.05) | 0.356 |
| **Ln triglyceride^b^** |  |  |  |  |  |  |
| Unadjusted model | **−0.604 (−0.848, −0.359)** | **<0.001** | **−0.019 (−0.031, −0.007)** | **0.002** | −0.003 (−0.008, 0.001) | 0.160 |
| Adjusted model 1**^c^** | **−0.611 (−0.859, −0.363)** | **<0.001** | **−0.019 (−0.031, −0.006)** | **0.003** | −0.003 (−0.008, 0.001) | 0.164 |
| Adjusted model 2^d^ | **−0.604 (−0.877, −0.330)** | **<0.001** | **−0.023 (−0.037, −0.009)** | **<0.001** | −0.003 (−0.008, 0.002) | 0.307 |
| **Total cholesterol** |  |  |  |  |  |  |
| Unadjusted model | **−0.60 (−0.95, −0.24)** | **0.001** | **−0.03 (−0.05, −0.01)** | **<0.001** | −0.002 (−0.009, 0.005) | 0.578 |
| Adjusted model 1**^c^** | **−0.54 (−0.90, −0.18)** | **0.003** | **−0.03 (−0.05, −0.01)** | **<0.001** | −0.002 (−0.008, 0.005) | 0.625 |
| Adjusted model 2**^d^** | **−0.55 (−0.95, −0.15)** | **0.007** | **−0.03 (−0.05, −0.01)** | **0.003** | −0.002 (−0.010, 0.005) | 0.497 |
| **Low-density lipoprotein** |  |  |  |  |  |  |
| Unadjusted model | **−0.48 (−0.78, −0.19)** | **0.001** | **−0.02 (−0.03, −0.005)** | **0.008** | −0.001 (−0.006, 0.005) | 0.747 |
| Adjusted model 1**^c^** | **−0.40 (−0.70, −0.10)** | **0.008** | **−0.02 (−0.03, −0.005)** | **0.010** | −0.0005 (−0.006, 0.005) | 0.848 |
| Adjusted model 2**^d^** | **−0.43 (−0.77, −0.09)** | **0.012** | **−0.02 (−0.04, −0.003)** | **0.017** | −0.001 (−0.007, 0.005) | 0.750 |
| **High-density lipoprotein** |  |  |  |  |  |  |
| Unadjusted model | **0.22 (0.09, 0.36)** | **0.001** | 0.001 (−0.006, 0.01) | 0.814 | 0.002 (−0.001, 0.004) | 0.206 |
| Adjusted model 1**^c^** | **0.20 (0.06, 0.33)** | **0.003** | 0.001 (−0.006, 0.01) | 0.840 | 0.001 (−0.001, 0.004) | 0.271 |
| Adjusted model 2**^d^** | **0.22 (0.08, 0.37)** | **0.003** | 0.003 (−0.004, 0.01) | 0.355 | 0.001 (−0.002, 0.003) | 0.568 |
| **Ln HOMA-IR^b^** |  |  |  |  |  |  |
| Unadjusted model | **−1.79 (−2.26, −1.31)** | **<0.001** | −0.015 (−0.039, 0.009) | 0.218 | −0.007 (−0.016, 0.002) | 0.143 |
| Adjusted model 1**^c,f^** | **−1.68 (−2.15, −1.20)** | **<0.001** | −0.014 (−0.038, 0.009) | 0.241 | −0.007 (−0.016, 0.001) | 0.097 |
| Adjusted model 2**^d,f^** | **−1.73 (−2.24, −1.22)** | **<0.001** | −0.019 (−0.044, 0.006) | 0.142 | −0.005 (−0.015, 0.004) | 0.279 |

Abbreviations: min, minutes; CI, confidence interval; HOMA-IR, insulin resistance from Homeostasis Model Assessment. β represents unstandardized regression coefficient. **^a^**Derived by dividing handgrip strength (kg) by body weight (kg). **^b^**Ln represents natural-log transformed outcome variables where the base of the log was 2.71828. **^c^**Adjusted for gender (when not stratified by gender), household wealth, and maternal education. **^d^**Additionally adjusted for sedentary time, time engaged in moderate-to-vigorous physical activity and awake wear time (all in minutes per day). **^e^**The models for association of standing long jump with systolic blood pressure were also adjusted for height (cm). **^f^**Additionally adjusted for sample storage time as it showed a negative correlation with fasting insulin level (Spearman’s ρ = −0.2, P<0.001). Statistically significant coefficients are presented in bold.
